# A Comprehensive and Effective Mass Spectrometry-Based Screening Strategy for Discovery and Identification of New Brassinosteroids from Rice Tissues

**DOI:** 10.3389/fpls.2016.01786

**Published:** 2016-11-30

**Authors:** Peiyong Xin, Jijun Yan, Bingbing Li, Shuang Fang, Jinshi Fan, Hailong Tian, Yong Shi, Weisheng Tian, Cunyu Yan, Jinfang Chu

**Affiliations:** ^1^National Center for Plant Gene Research (Beijing), Institute of Genetics and Developmental Biology, Chinese Academy of SciencesBeijing, China; ^2^College of Chemical Engineering, Qingdao University of Science and TechnologyQingdao, China; ^3^CAS Key Laboratory of Synthetic Chemistry of Natural Substances, Shanghai Institute of Organic Chemistry, Chinese Academy of SciencesShanghai, China

**Keywords:** brassinosteroids, discovery, identification, UPLC-MS, screening, biosynthetic intermediates, rice

## Abstract

The exploration and identification of new brassinosteroid (BR) compounds is critical to improve the biosynthetic research of BRs and expand the chemodiversity of active BRs. However, traditional methods are labor-intensive, time-consuming, and less sensitive. Here, we present a facile screening strategy for discovering and identifying novel BRs from plant tissues based on ultra-performance liquid chromatography-mass spectrometry (UPLC-MS). A total of 14 potential BRs were discovered from only 1 g of rice tissues and structurally elucidated by following a MS-based clue, acquired through multiple reaction monitoring (MRM) data-dependent enhanced product ion (EPI) scan, high resolution MS, and MS survey-dependent MS/MS. One of the 14 candidates was identified as 6-deoxo-28-homotyphasterol, a brand new BR compound that is reported for the first time in the BRs biosynthesis pathway. Detailed comparison with reference standards and quantitative level analysis in rice BR mutants confirmed the availability of the other candidates. This effective, yet simple method provides an efficient way to find more and more chemically new BR biosynthetic intermediates in plants, which is significant for complementing the biosynthesis and metabolism network of BRs. This strategy may also be used to discover unknown compounds of other plant hormone species as well as their key metabolites.

## Introduction

Brassinosteroids (BRs) are a polyhydroxylated steroid-type plant hormone with very similar structure to animal steroid hormones. They have well-known functions in various aspects of plant growth and development. BRs are involved in a variety of regulating processes including cell expansion, cell division and differentiation, photomorphogenesis, and skotomorphogenesis (Hayat, 2011). Furthermore, BRs have also been shown to be valuable in increasing crop productivity through genetic manipulation of BRs activity to alter plant metabolism and protect plants from environmental stresses (Sakamoto et al., 2006; Vriet et al., 2012). However, there still remains a lot of unknown and uncertain information about how BRs act at molecular level.

Elucidating the biosynthesis pathway of BRs is an important issue for various fields of BRs research (Fujioka and Yokota, 2003). However, the pathways remain not very clear. BRs are grouped into C_27_, C_28_, and C_29_ BRs according to the carbon numbers in their chemical structures. For C_28_ BRs, the profile of the network-like routes of BR biosynthesis has been described in great detail; however, the roles of some biosynthetic enzymes and their participation in the different subpathways still needs to be clarified (Fujioka and Yokota, 2003; Kim et al., 2008; Ohnishi et al., 2012). For C_27_ BRs and C_29_ BRs, which exhibit similar bioactivities to C_28_ BRs, the situation was even worse because little was known about their biosynthesis pathways (Fujita et al., 2006; Joo et al., 2015). Besides a lack of biochemical and enzymatic evidence about the biosynthetic pathways of C_27_ and C_29_ BRs, even most of the intermediate compounds on their pathways have not been explored and identified (Fujioka et al., 1999). In fact, the sum of C_27_ and C_29_ BRs found in previous reports amounts to only approximately 40% of the pool of all known BRs and intermediates (Hayat, 2011). The difficulty in discovering novel C_27_ and C_29_ BRs might be attributed to the lower endogenous levels of C_27_ and C_29_ BRs compared to C_28_ BRs, despite of higher levels of their biosynthetic precursors (Fujita et al., 2006; Ohnishi et al., 2006). Therefore, it is of the utmost priority to search for new unknown BRs to fill the gaps in the biosynthetic network.

In order to explore the occurrence of unknown BR intermediates in plants, a series of experiments have commonly been performed in previous studies. Dating back to the discovery of the first BR compound brassinolide (BL) from several tens of kilograms of pollens, it was first purified via successive chromatographic separations guided by bioactivity assays and then structurally identified through various approaches including NMR, MS, and X-ray crystal analysis. Finally, the bioactivity was further tested through the bean second-internode bioassay (Mitchell et al., 1970; Grove et al., 1979). During the following decades, this method was partially simplified to provide structural information only via gas chromatography-mass spectrometry (GC-MS) subsequent to derivatization (Kim et al., 1990; Fujioka et al., 1995; Hwang et al., 2006; Bajguz, 2009; Joo et al., 2015). The method was similar to or derived from the screening of bioactive natural products based on bioactivity-guided isolation and chemical screening (Hostettmann et al., 2001). In fact, this procedure proved effective and contributed a lot to the discovery of a large number of BR intermediates in different plant species. Nevertheless, there is no doubt that the method is less sensitive and consumes hundreds of grams to several kilograms of plant tissues, the collection of which is labor-intensive and very time-consuming. In addition, GC-MS usually cannot generate quasi-molecular ions or molecular ions, because target molecules are fragmented under 70 ev electron impact simultaneously with ionizing in the source. Thus, a sensitive, chemical structure-oriented, and high-performance MS-based screening and identification method needs to be developed.

Recently MS-based screening has been widely used to find target molecules containing identical molecular skeletons with different substituents in fields of drug metabolites, environmental contaminants, and natural products. In these studies, substructure-specific fragment ions or neutral losses were typically selected to guide a precursor ion or neutral loss scan-based screening process and the survey scan data-dependent MS/MS analysis was used for structural identification (Wen et al., 2008; Ni et al., 2010; Quadri et al., 2013; Hoff et al., 2014; Yan et al., 2014). However, these screening methods are not suitable for BRs due to the lower sensitivity of these survey scan modes in triple quadrupole MS and the extremely low endogenous BRs concentrations in plant tissues. Therefore, we focused on developing simple, selective, and high-efficiency sample pretreatment methods, while improving the ionization efficiency of BRs via derivitization for high-sensitivity detection and quantification of known BRs in previous studies (Xin et al., 2013a,b). In the present study, we tailored a sensitive, structure-oriented, and high performance MS-based strategy to screen and identify novel BRs from plant tissues based on multiple reaction monitoring (MRM) data-dependent enhanced product ion (EPI) scan merged with high resolution MS survey-dependent MS/MS analysis. With this strategy, 14 BRs were found in rice panicles and one candidate identified as 6-deoxo-28-homotyphasterol (6-deoxo-28-homoTY) using chemically synthesized standard, was discovered and reported for the first time. The effectivity and simplicity of this strategy makes it a promising method that will contribute to the biosynthetic study of BRs.

## Materials and methods

### Chemicals

CS (castasterone), TE (teasterone), and TY (typhasterol) were bought from Chemiclones, Inc. (Ontario, Canada). D_3_-6-deoxo-24-epiCS (D_3_-6-deoxo-24-epicastasterone), D_3_-CS, 6-deoxo-24-epiCS, 24-epiBL (24-epi-brassinolide), and 24-epiCS (24-epicastasterone) were from OlChemIm Ltd. (Olomouc, Czech Republic). 28-homoBL (28-homo-brassinolide) was obtained from Vegcides Bio-Farm Co., Ltd. (Shanghai, China). DMAPBA [3-(dimethylamino)-phenylboronic acid] was purchased from TCI Development Co. Ltd. MeOH and ACN (HPLC grade) were obtained from Fisher Scientific. Mixed anion exchange (MAX) and mixed cation exchange (MCX) cartridges (6 mL and 500 mg) were purchased from Waters Company. Deionized water purified by the Elga Purelab water purification system (resistivity >18.2 MΩ) was used for all experiments.

### Plant materials

Wild-type *Nipponbare* (Nip), *Shiokari* (Shio), *TC65*, and *d61-2*, M107, *d2-2* mutants of rice (*Oryza sativa*) were cultured at a farm located in Changping district of Beijing, China and harvested during flowering. For screening of potential BRs, the panicles including glumes and rachis-branches were isolated, collected, and quickly frozen in liquid nitrogen for further processing for BRs purification.

### Purification and derivatization of BR candidates from plant materials

The collected panicles were first ground to a fine powder with a MM-400 mixer milling (Retsch). One gram of the plant material powder was extracted twice, using 6.25 mL of 95% (v/v) aqueous MeOH in total. For quantification of the BR candidates, D_3_-6-deoxo-24-epiCS (500 pg) and D_3_-CS (1 ng) were added to the powder as internal standards (IS) and were subjected to extraction parallelly.

The BR candidates were purified according to a protocol developed in our lab as previously reported, but with minor modifications (Xin et al., 2013b). The MAX cartridge was activated and equilibrated with MeOH, water, 1 M KOH, 10% (v/v) MeOH, and 95% (v/v) MeOH in turn, and the MCX cartridge with MeOH, water, 5% (v/v) formic acid (FA), and 10% (v/v) MeOH. Then, the crude plant extracts were passed through a MAX column and collected to be dried by N_2_ chilling. After reconstructed in 4 mL of 10% (v/v) MeOH, the extracts were loaded onto the equilibrated MCX cartridge. After sequential washing with 5% (v/v) FA in 5% (v/v) MeOH, 5% (v/v) MeOH, 5% (v/v) NH_4_OH in 5% (v/v) MeOH, and 5% (v/v) MeOH, the fraction containing BR candidates was eluted with 95% (v/v) MeOH. The elution was dried and then dissolved in 200 mL of ACN for derivatization with 30 mg DMAPBA.

### Non-targeted screening of BR candidates in rice panicles on hyphenated UPLC-QTrap MS and UPLC-QTof MS systems

The screening process was performed on a hybrid quadrupole linear ion trap MS (QTRAP 5500, AB SCIEX) equipped with an electrospray ionization (ESI) source coupled with a UPLC (Waters). Five microliters per sample were injected onto a BEH C18 column (100 × 2.1 mm, 1.7 μm). To obtain maximal coverage upon BR candidates, the inlet method was also adjusted as following: mobile phase A, 0.05% (v/v) acetic acid in water and B, 0.05% (v/v) acetic acid in ACN. Gradient profile: 0 to 17.5 min, 70% B to 95% B; 17.5 to 19.0 min, 95% B; 19.0 to 20.5 min, 95% B to 70% B; and 20.5 to 23.5 min, 70% B. ESI source parameters were set as: ion spray voltage, 5500 V; desolvation temperature, 550°C; nebulizing gas 1, 45; desolvation gas 2, 45; and curtain gas, 27.

When performing MRM screening, Q3 masses were set to 190.1 and 176.1, while Q1 masses were set to all even numbers ranging from 548.4 to 624.4 according to the known BRs structure reported in the literature (Hayat, 2011). The dwell time of each channel was set to 5 ms and the interchannel pause time was 5 ms. N_2_ was used as collision gas and the collision energies for [M+H]^+^ > 190.1 and [M+H]^+^ > 176.1 were 55 V and 70 V, respectively. Both Q1 and Q3 were operated at unit resolution.

When performing MRM-dependent EPI acquisition, the MRM transitions screened out were directly transferred to the MRM survey part of the MRM-IDA(information dependent acquisition)-EPI method. Criteria for IDA-EPI were set for 1–2 most intense ions in each dynamic background subtracted MRM survey scan spectrum with an intensity threshold of 1000 counts per second (CPS). The mass range of the EPI scan ranged from 100 to 650 Da and scan speed was set as 10,000 amu/s. Collision energy (CE) was set to 70 V with a spread at 5 V and the Q3 linear ion trap (LIT) was dynamically filled to avoid overfilling. The time duration of each MRM-IDA-EPI cycle was 0.8–1.0 s.

When performing accurate mass and MS/MS determination of BRs candidates, a high resolution MS scan-dependent MS/MS acquisition method was developed on a UPLC-QTof-MS (Synapt G2 HDMS, Waters) system equipped with an ESI source. The UPLC method was set exactly same to the above description. The source parameters were set as follows: capillary voltage, 3000 V; sampling cone voltage, 40 V; extraction cone voltage, 4 V; source temperature, 100°C; desolvation temperature, 350°C; cone gas, 50 L/h; and desolvation gas, 800 L/h. To ensure the accurate mass measurement, Leu-enkephalin was used as lock mass in MS/MS mode (m/z 278.1141 and 556.2771 for positive ion mode) at a concentration of 200 pg/μL with a flow rate at 10 μL/min. All experiments were performed in positive resolution mode to provide a resolution at 20,000. The m/z of the BR candidates found by MRM-IDA-EPI was input into the including masses list and the occurrence of these ions in the MS spectrum would trigger auto MS/MS scans. For MS/MS acquisition, argon was used as collision gas and the collision energy was ramped up from 45 to 55 eV. The mass ranges of the MS survey scan and the dependent MS/MS scan were 500–650 and 100–650 Da, respectively. The cycle time was 0.6 s, consisting of 200 ms for MS acquisition and 400 ms for MS/MS acquisition.

The molecular formulae of the BR candidates were generated from the accurate masses obtained from the MS scan via the Elemental Composition function of Masslynx software. The results were generated with a mass tolerance within 3 ppm (mass error, Δm/m), and three peaks were used to generate an isotope fit value. The double-bond equivalent (DBE) value was set to 4–15. The element limits were set as follows: C, 30–40; H, 45–65; O, 3–8; N, 1–2; and B, 1–2.

### Semiquantitative analysis of BR candidates in mutant plants

The endogenous levels of BR candidates in mutant plants were evaluated by the ratio of the integrated peak area of these compounds to that of the D-labeled internal standards (S/S_IS_), which might be described as the relative abundance to IS. The closer one of either D_3_-6-deoxo-24-epiCS or D_3_-CS to the candidate compound in retention time would be selected as the IS for it.

## Results and discussion

### Fragmentation profile/pattern of derivatized BRs via collision-induced dissociation

**Figure 1:**
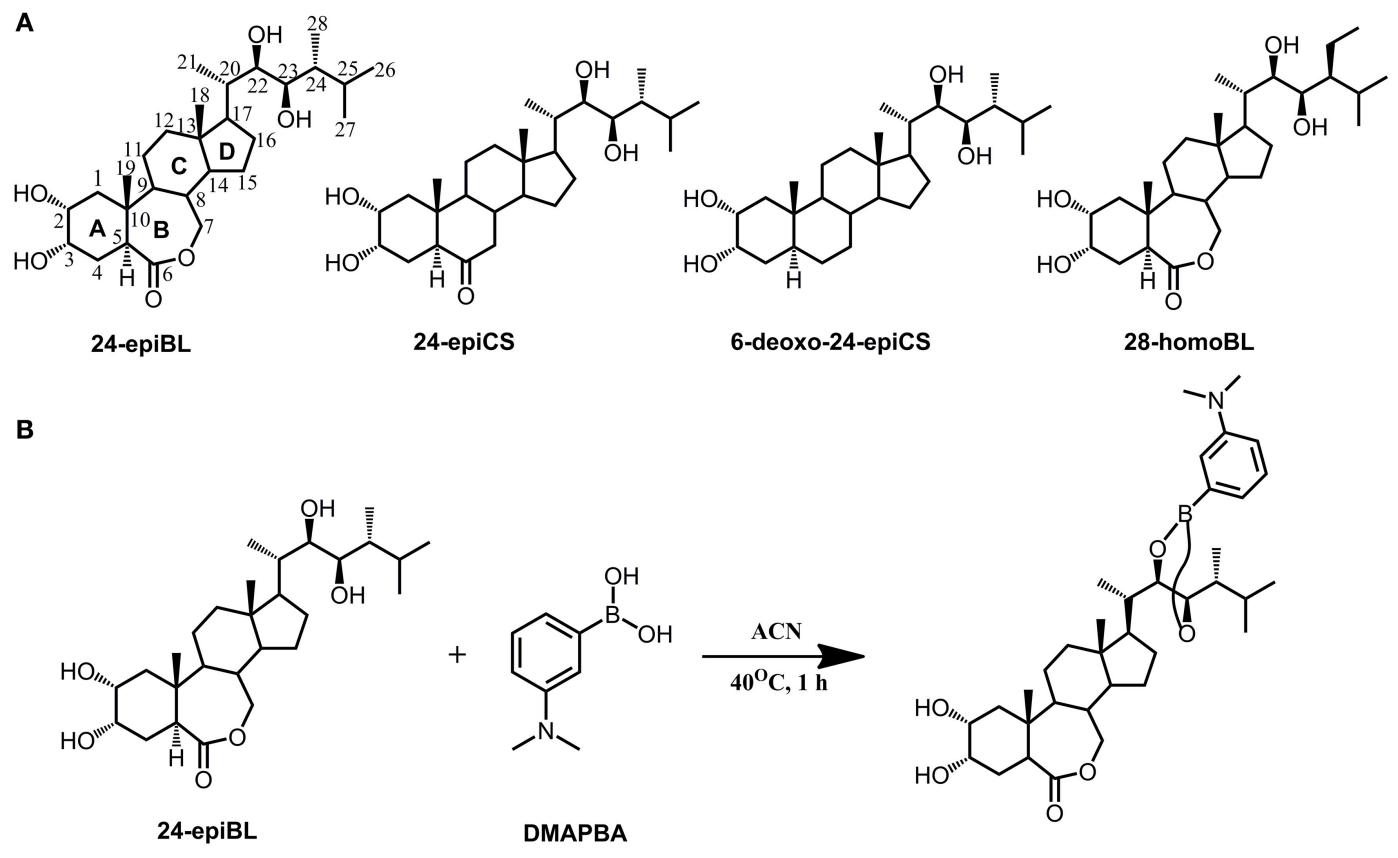
**Chemical structures of representative BRs and derivatization with DMAPBA. (A)** Chemical structures of representative BRs. Using 24-epiBL as example, all BRs are featured with a four-ring skeletal structure (marked as A, B, C, and D ring) connected with a side chain. The structural variances in B ring and side chain determine the BRs type and all the carbon atoms are numbered in sequence. C_27_, C_28_, and C_29_ BRs are grouped according to the carbon number of different substituent groups on the C_24_ site. **(B)** Representative derivatization reaction formula of 24-epiBL with DMAPBA.

**Figure 2:**
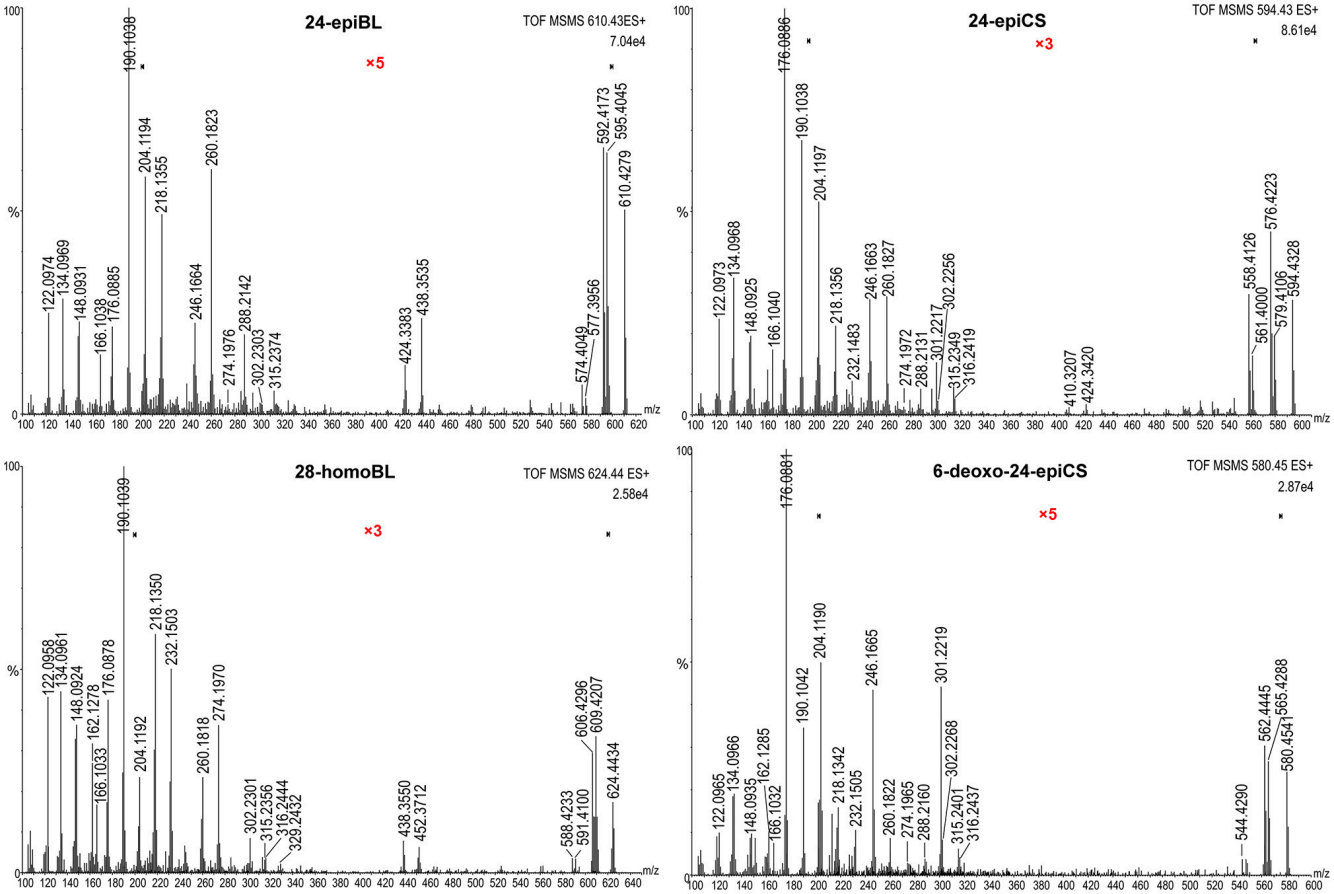
**High-resolution MS/MS spectra of DMAPBA-derivatized representative BRs**. All spectra were obtained on Synapt G2 QTof-MS with a resolution of 20,000. The spectra were combined with 10 MS/MS full scans. The Y-axis represents the relative abundances of ions in %. The magnified regions were marked as “x3” or “x5” colored in red.

The fragmentation pattern of known BRs was an important clue to follow to discover and identify novel potential BR candidates with MS-based techniques. Therefore, we first investigated the fragmentation behavior of BRs with representative chemical structures under collision-induced dissociation (CID) conditions performed on a QTof-MS instrument, capable of producing high-resolution (HR) and accurate mass to charge ratio (m/z) MS/MS data. Due to the common occurrence of vicinal C_22, 23_ diol groups in more than 80% known BR compounds, our study was focused on C_22, 23_ diol-BRs. Four BRs including 24-epiBL, 24-epiCS, 6-deoxo-24-epiCS, and 28-homoBL were chosen as representatives of B-ring lactone-type C_28_ BRs, B-ring cycloketone-type C_28_ BRs, 6-deoxo C_28_ BRs and B-ring lactone-type C_29_ BRs, respectively (Figure 1A). Their 24-epi isomers BL, CS, and 6-deoxoCS were unavailable although these are major BRs in plants. To enhance their MS responses in ESI mass spectrometry, BRs were derivatized with DMAPBA through formation of boronate esters with vicinal C_22, 23_ hydroxyl groups on the side chain (Figure 1B). The HR MS/MS spectra of derivatized BR standards are shown in Figure 2 and the characteristic fragment ions are summarized in Table 1.

**Table 1:** **Characteristic product ions and their relative abundances in high-resolution MS/MS of DMAPBA-derivatized representative BRs**.

	**24-epiBL**	**24-epiCS**	**28-homoBL**	**6-deoxo-24-epiCS**
Precursor ion	610.4279	594.4328	624.4434	580.4541
	50.3%	28.3%	17.3%	26.4%
A ring structure-related ions	592.4173	576.4223	606.4296	562.4445
	13.1%	45.1%	9.8%	7.9%
	574.4049	558.4126	588.4233	544.4290
	1.5%	9.9%	1.2%	0.7%
B ring structure-related ions	424.3383	424.3420	438.3550	
	2.4%	0.9%	2.6%	
	438.3535		452.3712	
	4.7%		2.1%	
D ring structure-related ions	301.2213	301.2217	315.2356	301.2223
	0.6%	4.3%	1.1%	8.9%
	302.2303	302.2256	316.2444	302.2268
	0.5%	1.1%	1.2%	2.4%
	315.2374	315.2349	329.2432	315.2401
	0.5%	2.3%	0.7%	1.4%
	316.2411	316.2419		316.2437
	0.4%	1.3%		1.0%
Side chain structure-related ions	176.0885	176.0886	176.0878	176.0881
	21.6%	100%	42.5%	100%
	190.1038	190.1038	190.1039	190.1042
	100%	67.6%	100%	34.5%
	204.1194	204.1197	204.1192	204.1190
	11.7%	17.5%	7.8%	10.6%
	218.1355	218.1356	218.1350	218.1342
	9.8%	7.3%	19.6%	3.8%
	232.1436	232.1483	232.1503	232.1505
	0.5%	1.6%	16.7%	2.2%
	246.1664	246.1663	246.1697	246.1665
	4.5%	9.5%	0.8%	8.8%
	260.1823	260.1827	260.1818	260.1822
	12.1%	9.7%	7.8%	2.7%
	274.1976	274.1972	274.1970	274.1965
	0.5%	0.7%	12.1%	0.6%
	288.2142	288.2131	288.2119	288.2160
	3.9%	2.1%	0.6%	0.8%
			302.2301	
			2.8%	

All MS/MS spectra of the four representative BRs could be divided into three regions based on their fragmentation pathways. In the high mass region from 50 Da below the m/z_[M+H]+_ to m/z_[M+H]+_, the MS/MS spectra were dominated by ions produced through neutral loss of H_2_O, CH_3_·. In addition, the number of the continuous neutral H_2_O losses indicated the number of hydroxyl groups located on the A ring of BRs. Thus, this m/z region was an indicator of A ring structure. The abundance of [M+H-H_2_O]^+^ was dependent on the stability of the product ions, and was fundamentally decided by the B ring structure and the stereochemistry of C_3_-OH. In the low-mass region below about 300 Da, the fragment ions were rather abundant, the majority of which were fragments of the derivatization reagent DMAPBA itself and DMAPBA connected with the side chain fragments. The ion at m/z 166.10 could be assigned to the fragment of DMAPBA (C_8_H_12_BNO_2_, m/z = 166.1039). The product ions below m/z 166.10 were mainly related to fragments of DMAPBA, which were independent of BRs structure and contributed little to the identification of BRs. The ion at m/z 190.10 (C_10_H_12_BNO_2_, m/z = 190.1039) originated from the vicinal C_22_, C_23_ hydroxyl groups, derivatized by DMAPBA and the neutral loss of -CH_2_ from the ion at m/z 190.10 generated the ion at m/z 176.09. The ion set at m/z 190.10, 204.12 (C_11_H_14_BNO_2_, m/z = 204.1196), 218.14 (C_12_H_16_BNO_2_, m/z = 218.1352), 246.17 (C_14_H_20_BNO_2_, m/z = 246.1665), 260.18 (C_15_H_22_BNO_2_, m/z = 260.1822), and 288.21 (C_17_H_26_BNO_2_, m/z = 288.2135) was from the side chain fragments linked with different −CH_2_ units. Therefore, the highest m/z of this series indicated the carbon number of BRs because of the diversity at the C_24_ site. In the median mass region ranging from approximately 300 to 500 Da, the fragment ions could rarely be found but very important for BRs structure elucidation because these ions were produced through opening of B ring and D ring. However, different fragment patterns occurred between lactone-type BRs and cycloketone-type, 6-deoxo-type BRs. For lactone-type BRs, such as 24-epiBL and 28-homoBL, the relative intensities of fragment ions at m/z 424.34 and 438.36 (C_28_ BRs) or m/z 438.36 and 452.37 (C_29_ BRs) resulting from dissociation of the seven-member B ring, were higher than the fragment ions at m/z 301.22 and 315.24 (C_28_ BRs) or m/z 315.24 and 329.25 (C_29_ BRs) that resulted from the cross-ring cleavage of the D ring. However, cycloketone-type and 6-deoxo-type BRs turned out to be contradictory. This phenomenon could be attributed to the heterolysis of C-O bonding being easier to occur than the homolysis of C-C bonding under CID conditions. Furthermore, the mass difference of 14.016 Da between the two ion series of C_28_ and C_29_ BRs also indicated the carbon number of BRs. It was obvious that the corresponding ions for C_27_ BRs were at m/z 410.32 and 424.34.

To summarize (Table 1), the matching between the characteristic daughter ions and the BRs structure characteristics could be described as follows: First, the occurrence of product ions at m/z 176.09 and 190.10 suggested the vicinal diol groups located at C_22_ and C_23_ sites. Next, the number of continuous neutral H_2_O losses in the high-mass region was related to the number of hydroxyl groups on the A ring. Moreover, the relative abundances of ions at m/z 424.34, 438.36 (C_28_ BRs) or m/z 438.36 and 452.37 (C_29_ BRs) and m/z 301.22, 315.24 (C_28_ BRs) or m/z 315.24 and 329.25 (C_29_ BRs) indicated the structural type of the B ring and the carbon number of BRs. Last, the ion series from m/z 190.10 to 288.21 indicated the side chain and the highest mass of this series was also correlated with the carbon number.

### Workflow design for the discovery of potential BRs in plant tissues

**Figure 3:**
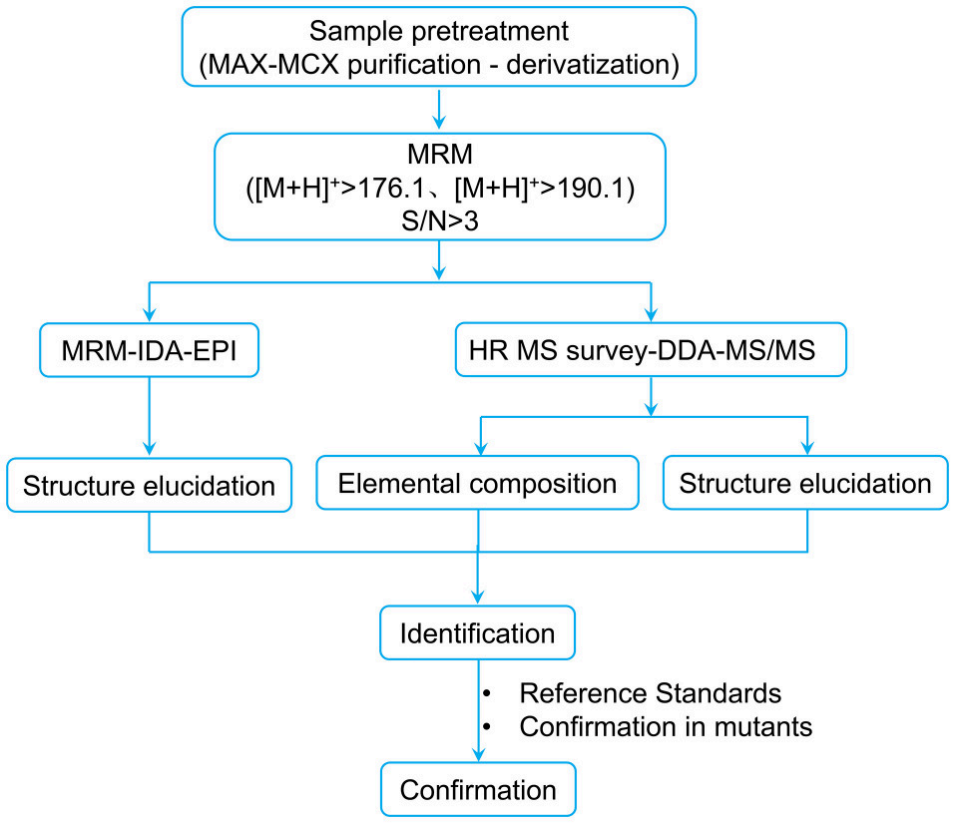
**UPLC-MS-based screening workflow for discovering potential BRs from plant tissues**. DDA refers to data dependent acquisition.

Taking the point-to-point correlation between BRs structures and the MS/MS characteristics into account, a comprehensive UPLC-MS-based screening workflow was established to search for potential BRs in rice panicle extracts (Figure 3).

In previous reports that were involved in non-target MS-based screening for bioactive compounds, neutral loss or precursor ion scan based on loss of a neutral characteristic substructure or generation of a characteristic daughter ion with a specific substructure was commonly used on triple quadrupole MS systems. At this time, quadrupoles were used in mass scanning mode acquiring full scan mass spectra resulting from the DC (direct current) and RF (radio frequency) voltages ramping process. Therefore, the sensitivity and scanning speed of precursor ion scan and neutral loss scan are far lower than MRM mode in which quadrupoles work in filtering mode. Hereby, a fixed set of DC and RF voltages is applied to Q1 and Q3 and thus only a single m/z can pass while ions with different m/z are filtered out.

**Figure 4:**
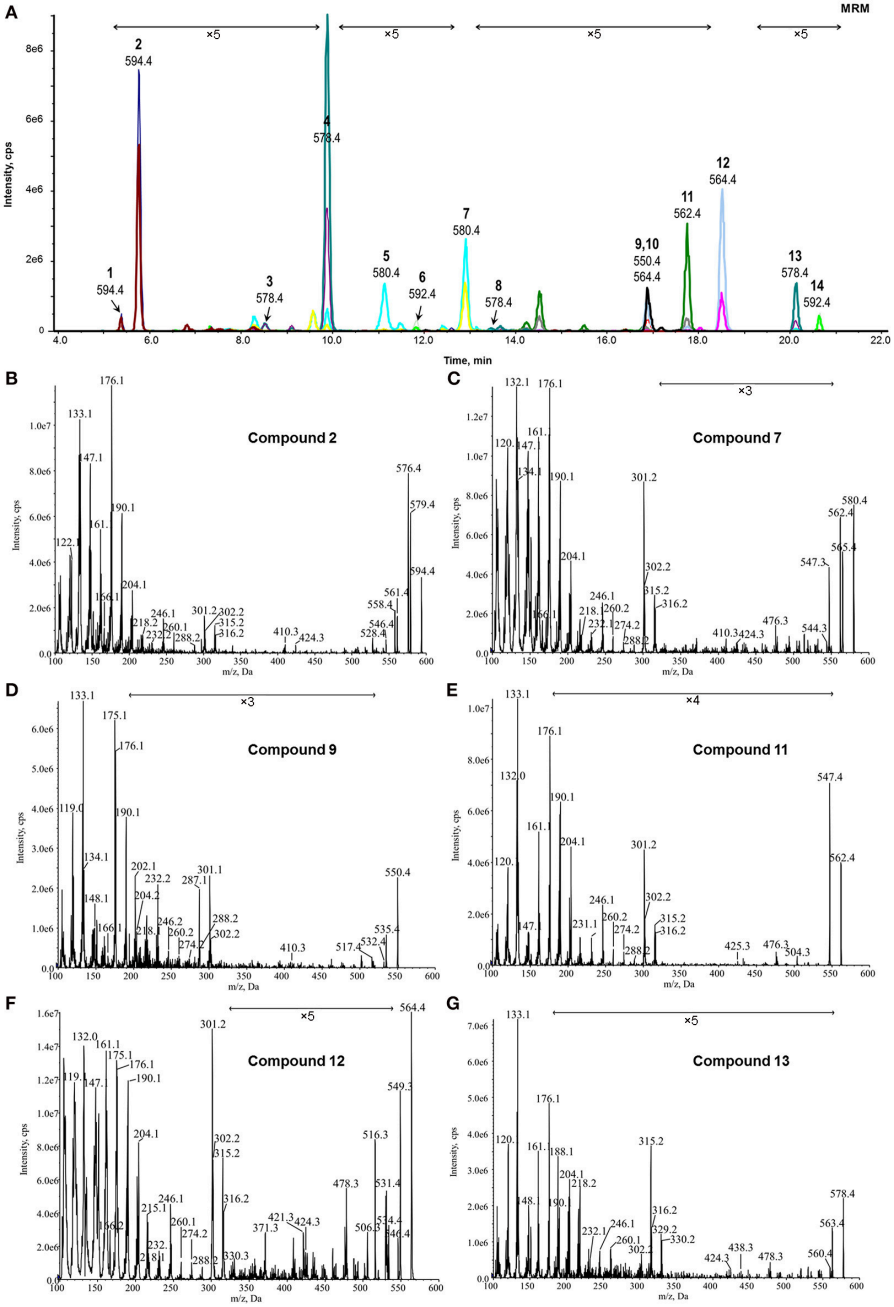
**MRM-dependent EPI analysis of potential BRs from plant tissues. (A)** Overlapped MRM chromatograms of potential BRs; **(B–G)**, Representative MRM-dependent EPI spectra of candidate BR compounds 2, 7, 9, 11, 12, and 13. The Y-axis represents the relative abundances of ions in CPS.

Because BRs are present in plants at an extremely low concentration, neutral loss scan or precursor ion scan mode was not suitable for BRs screening for sensitivity reasons, and thus, a MRM-based screening method was considered for the discovery of unknown BRs although it is often used for quantitation of known compounds. As is well-known, a combination of precursor and product ions is needed to create any MRM method. Therefore, the ions at m/z 176.09 and 190.10 were chosen as diagnostic product ions. Although the precursor ions were unknown, we proposed all even-numbered ions covering from the known lowest mass of 548.4 Da to the known highest mass of 624.4 Da as the hypothetical precursors benefitted from the fast scanning speed of QTrap mass spectrometry. Finally, a MRM-based pre-survey process was performed using the transitions [M+H]^+^ > 190.1 and [M+H]^+^ > 176.1 as diagnostic channels, with [M+H]^+^ set as any even number between 548.4 and 624.4. CE as a critical parameter was set according to the optimized CE of 6-deoxo-24-epiCS, considering its median polarity among all known BRs. The overlapped MRM chromatographic profiles are shown in Figure 4A. From the overall 78 transitions monitored, 32 precursors detected with both of the [M+H]^+^ > 190.1 and [M+H]^+^ > 176.1 MRM channels at the same retention time in a single LC-MS/MS run were reserved as candidates subject to second-round screening.

The reserved 32 precursors were then transferred to a MRM-dependent parallel EPI scan acquisition method to obtain more structural information of these specific candidates. Although the combination of UPLC and sub-2 μm packings could provide a high column efficiency and powerful separation, some poor-resolved precursor peaks still remained in the MRM chromatogram (Figure 4A). Thus, to prevent that key candidates were missed, an EPI scan was carried out for the two most abundant ions in each dynamic background subtracted MRM survey scan spectrum with an intensity threshold of 1000 CPS considering the noise level. In the combined EPI spectrum of each candidate (Figures 4B–G, Figures S1, S2), the presence of the ion series from m/z 176.1 to m/z 316.1 was used as the criteria to recognize a candidate as a potential BR. Twenty-one precursors met the criteria and were reserved for further identification. The remaining precursors without this feature or producing poor-quality EPI spectra were eliminated from the candidate list.

**Figure 5:**
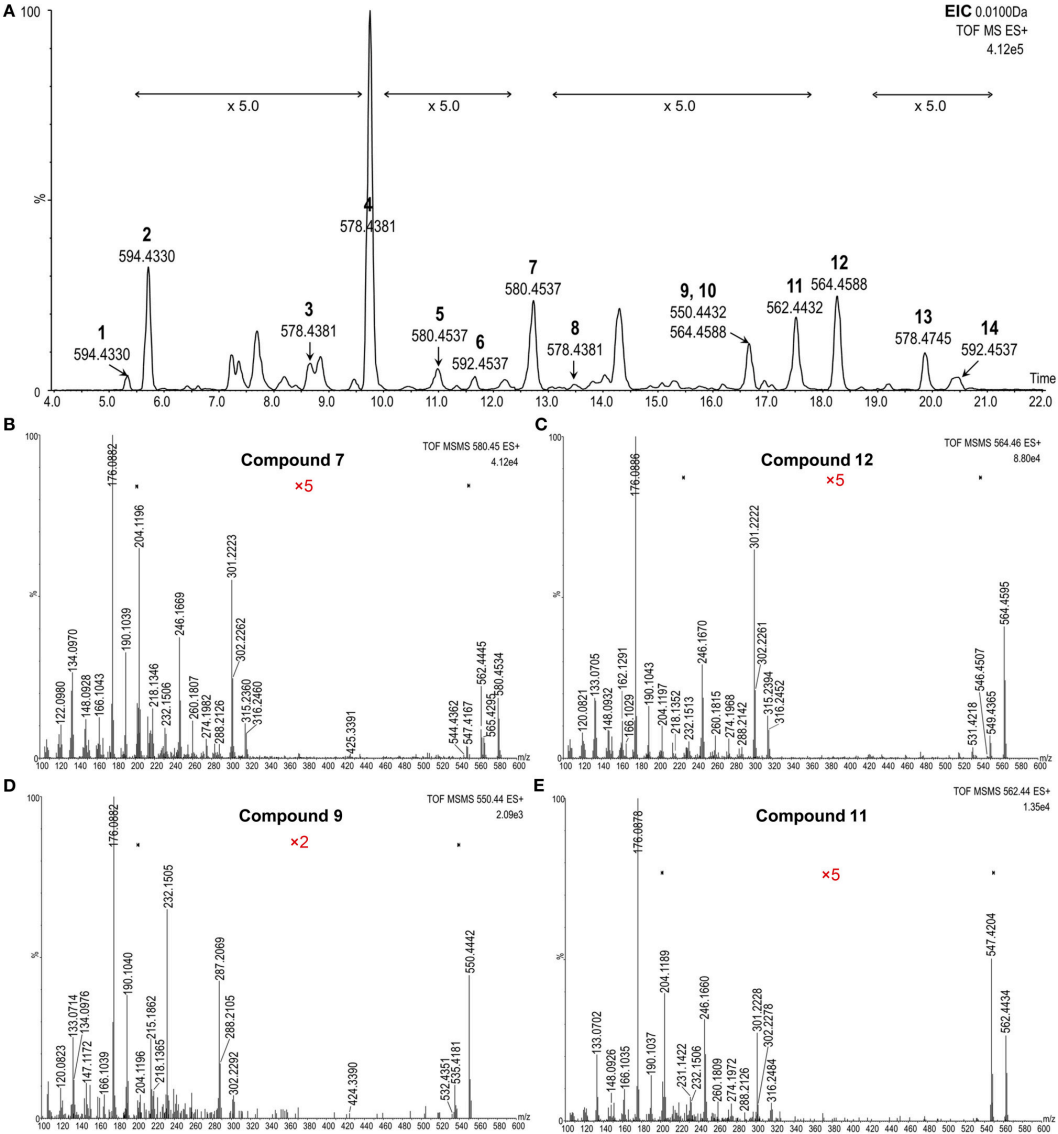
**MS survey-dependent HR MS/MS analysis of BR candidates from plant tissues. (A)** Extracted ion chromatograms (EICs) of potential BRs; **(B–E)**, Representative MS survey-dependent HR MS/MS spectra of candidate compounds 7, 12, 9, and 11. The Y-axis represents relative abundances of ions in %.

To further confirm these precursors, a high resolution and accurate MS survey-dependent MS/MS acquisition method was established on a hyphenated UPLC-QTof-MS system. From the MS survey spectrum generated from the extracted ion chromatogram (EIC) (Figure 5A), the molecular formulae of these precursors could be calculated from their accurate masses with a mass tolerance within 3 ppm considering the resolution setting of the instrument. Additionally, the characteristic boron isotopic patterns of the [M+H]^+^ peaks were used as another criteria to detect a potential BR. In the high resolution MS/MS spectrum (Figures 5B–E, Figures S3, S4C), the mass tolerance of the proposed diagnostic daughter ions was set to <10 ppm compared to theoretical values. All candidates violating these rules were excluded. Due to the lower sensitivity of the QTof-MS system than QTrap-MS and the extremely low concentration of some compounds, the product ions at m/z 410.32, 424.34, 438.36, and 452.37 might be absent in the MS/MS spectrum and were not monitored here. Eventually, only 14 compounds went through the screening process and their information is summarized in Table 2.

**Table 2:** **Summarized information of 14 BR candidates**.

**Co.ID**	**Theoretical m/z**	**Measured m/z and error (ppm)**	**Derivatized formula**	**Native formula**	**Name**	**RT (min)**	**Isotopic pattern (%)**	**B ring characteristics (S/N of 410.3, 424.3, 438.3, 452.3 channel)**
1	594.4330	594.4315	C_36_H_56_BNO_5_	C_28_H_48_O_5_	Isomer of CS	5.38	15.94	3, n.d., 4, n.d.
		−2.5				5.33	41.57	
2^*^	594.4330	594.4333	C_36_H_56_BNO_5_	C_28_H_48_O_5_	CS	5.76	21.05	25, 39, 18, 4
		0.5				5.70	39.24	
3^*^	578.4381	578.4396	C_36_H_56_BNO_4_	C_28_H_48_O_4_	TE	8.53	25.09	n.d., 4, 3, n.d.
		2.6				8.47	35.56	
4^*^	578.4381	578.4376	C_36_H_56_BNO_4_	C_28_H_48_O_4_	TY	9.88	21.48	111, 158, 103, 27
		−0.9				9.80	36.11	
5	580.4537	580.4545	C_36_H_58_BNO_4_	C_28_H_50_O_4_	Isomer of 6-deoxo-CS	11.14	21.1	n.d., 7, 6, n.d.
		1.4				10.97	32.61	
6	592.4537	592.4529	C_37_H_58_BNO_4_	C_29_H_50_O_4_	28-homoTY	11.83	23.23	n.d., n.d., 6, 7
		−1.4				11.66	35.42	
7	580.4537	580.4540	C_36_H_58_BNO_4_	C_28_H_50_O_4_	6-deoxo-CS	12.92	25.21	48, 40, 38, 8
		0.5				12.74	37.07	
8	578.4381	578.4387	C_36_H_56_BNO_4_	C_28_H_48_O_4_	Isomer of TY and TE	13.49	22.18	12, 24, 11, 116
		1.0				13.42	34.10	
9	550.4432	550.4432	C_35_H_56_BNO_3_	C_27_H_48_O_3_	6-deoxo-28-norTY/TE	16.90	22.07	7, 6, n.d., n.d.
		0.0				16.66	37.28	
10	564.4588	564.4601	C_36_H_58_BNO_3_	C_28_H_50_O_3_	6-deoxo-TE	16.87	20.40	3, 4, 6, n.d.
		2.3				16.64	29.57	
11	562.4432	562.4432	C_36_H_56_BNO_3_	C_28_H_48_O_3_	6-deoxo-DT	17.76	20.98	7, 8, 4, n.d.
		0.0				17.51	37.22	
12	564.4588	564.4598	C_36_H_58_BNO_3_	C_28_H_50_O_3_	6-deoxo-TY	18.52	20.99	74, 127, 112, 23
		1.8				18.27	35.83	
13^*^	578.4745	578.4752	C_37_H_60_BNO_3_	C_29_H_52_O_3_	6-deoxo-28-homoTY	20.14	21.52	3, 3, 19, 13
		1.2				19.70	36.15	
14	592.4537	592.4537	C_37_H_58_BNO_4_	C_29_H_50_O_4_	Isomer of 28-homoTY	20.64	19.79	n.d., n.d., n.d., n.d.
		0.0				20.38	39.81	

The relative mass errors in ppm are calculated via [(m/z_measured_ – m/z_theoretical_)/m/z_theoretical_]. RT refers to retention time and n.d. refers to not detectable. The two values of every compound in the column of RT stand for the retention times on UPLC-QTrap-MS (upper) and UPLC-QTof-MS (lower) respectively. The two values of the isotopic pattern represent relative abundances of ^10^B^12^C-BRs and ^11^B^13^C-BRs to ^11^B^12^C-BRs, respectively. Compounds marked with ^“*”^ were identified, while others were not identified.

From the screening process described above, we can draw the conclusion that this strategy can be extended to investigate other plant hormones. For example, all cytokinins (CKs) share a common purine skeleton and product ion at m/z 136.06 in MS/MS (Sakakibara, 2006); therefore, the method may be transferred to find new CKs compounds. More interestingly, strigolactones (SLs) are a novel type of plant hormone and the biosynthetic research of SLs is still in the early stages of development and has been a spotlight for phytologists in recent years (Al-Babili and Bouwmeester, 2015). Compared with BRs and CKs, all discovered SLs possess a five-numbered D ring and generate a subsequent product ion at m/z 97.03 in MS/MS, which is a favorable preference for the use of our strategy. Thus, our method is promising for playing an important role in the research of other plant hormones.

### Structure elucidation of representative potential BRs and confirmation with authentic standards

**Figure 6:**
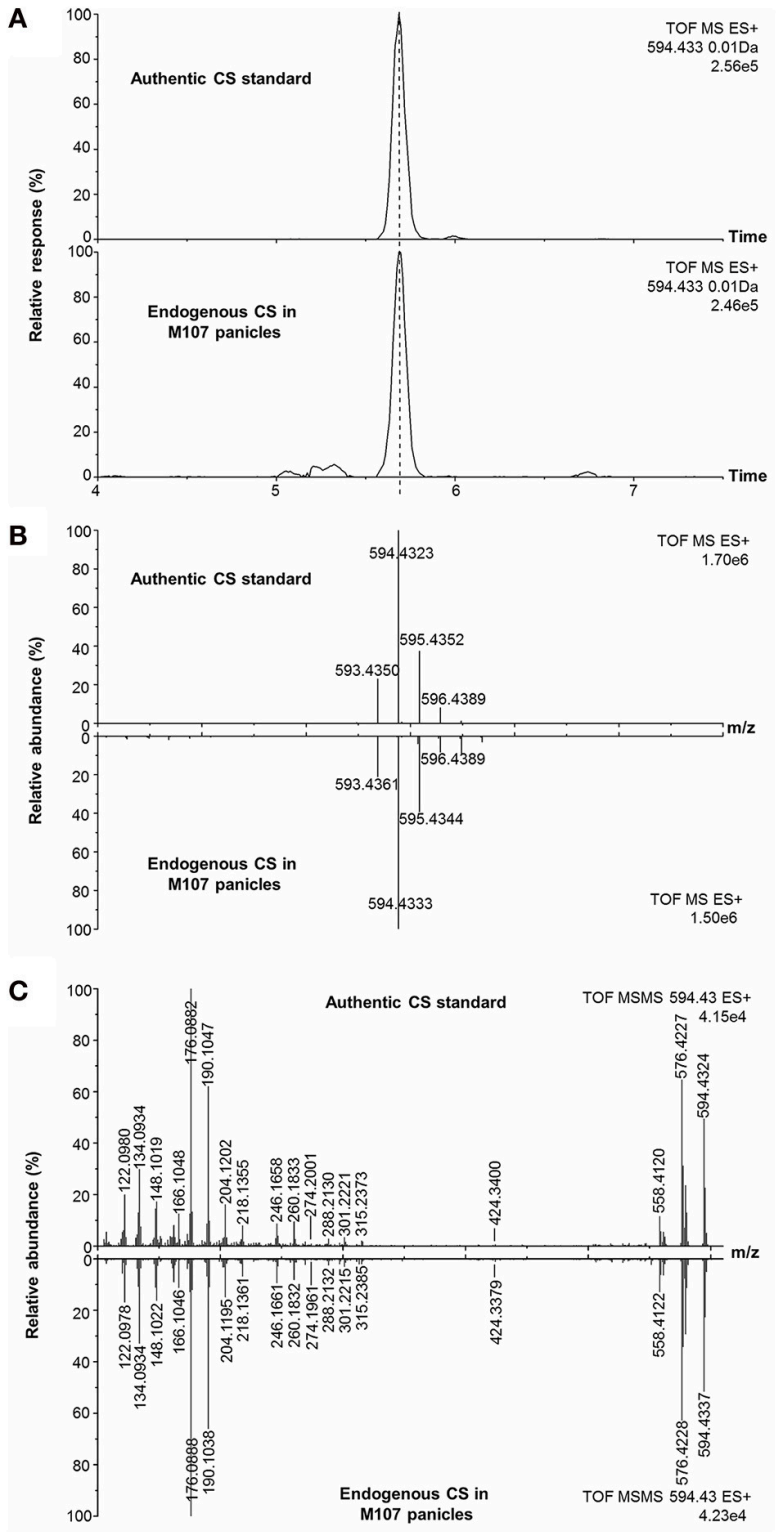
**Identification of endogenous CS in plant tissues with reference standard. (A)** EIC of endogenous CS and CS reference standard; **(B)** Isotopic pattern of endogenous CS and CS reference standard; **(C)** High-resolution MS/MS spectrum of CS reference and endogenous CS.

According to the obtained comprehensive MS-based information, the chemical structures of the potential BRs screened out were predicted. The measured m/z of [M+H]^+^ of compound 2 was 594.4333 and the molecular formula was calculated to be C_36_H_56_BNO_5_ (theoretical m/z = 594.4330, 0.5 ppm) (Figure 6B). After deducting the contribution of the derivatization reagent DMAPBA, the native formula was calculated as C_28_H_48_O_5_, which was identical to CS. The [M+H]^+^ peaks showed an apparent characteristic boron isotopic abundance distribution, which was 21.48, 100, and 36.11% in contrast to the theoretical distribution (22.59, 100, and 38.52% for ^10^B^12^C-CS, ^11^B^12^C-CS, and ^10^B^13^C-CS, respectively) with relative deviations at −4.91, 0, and −6.26% (Figure 6B). This particular result indicated the existence of C_22_, C_23_-diol substructure in compound 2. The EPI spectrum contained product ions at m/z 576.4 and m/z 558.4 generated from consecutive losses of H_2_O, which suggested that two hydroxyl groups were located on the A ring (Figure 4B). This result was confirmed by the accurate masses of both ions obtained on a QTof-MS system, measured as 576.4228 (theoretical m/z = 576.4224, Δm/m = 0.7 ppm) and 558.4122 (theoretical m/z = 558.4119, Δm/m = 0.5ppm) (Figure 6C). The product ions at m/z 301.2, 302.2, 315.2, and 316.2 were much more abundant than m/z 410.3, 424.3 in the MS/MS spectra (Figures 4B, 6C). This phenomenon combined with the molecular formula information and the hydroxyl group number on A ring and the side chain leads to the conclusion that B ring of this compound was cycloketone-structured. The EPI spectrum and the high resolution MS/MS spectrum further revealed that both of them contained the ion series from m/z 190.10 to 288.21, a typical MS/MS characteristic of the BRs side chain. In addition, the S/N ratios of MRM transitions [M+H]^+^ > 410.4, 424.4, 438.4 were 4.5-fold higher than the transition [M+H]^+^ > 452.4, providing further evidence for the C_28_ structuring of the compound (Table 2). To summarize from these MS-based information points, compound 2 was revealed to be a C_28_ cycloketone-type BR with two hydroxyls on the A ring and another two vicinal ones on the side chain and could be easily recognized to be CS or its isomer. A detailed comparison with the authentic CS standard in the retention time as well as MS and MS/MS behaviors (Figures 6A–C, Table 3), compound 2 was confirmed as CS. Similarly, compound 3 and 4 were identified as TE and TY (Figure S4, Table 3).

**Table 3:** **Comparison of chromatographic, MS, and MS/MS properties between endogenous BR candidates and authentic standards**.

	**CS**		**TY**		**6-deoxo-28-homoTY**	
	**Authentic**	**Endogenous**	**Authentic**	**Endogenous**	**Authentic**	**Endogenous**
R_*t*_ (min)	5.70	5.70	9.80	9.80	19.67	19.67
Isotopic pattern of [M+H]^+^	593.4350 (22.9%)	593.4361 (21.0%)	577.4402 (23.0%)	577.4396 (21.5%)	577.4771 (23.1%)	577.4781 (22.4%)
	594.4323 (100%)	594.4333 (100%)	578.4371 (100%)	578.4376 (100%)	578.4742 (100%)	578.4752 (100%)
	595.4352 (37.3%)	595.4344 (39.2%)	579.4404 (37.1%)	579.4396 (36.1%)	579.4771 (38.7%)	579.4786 (36.0%)
Characteristic fragment ions (m/z) and relative abundance (%)	122.0980 (19.9%) 134.0934 (29.8%) 148.1019 (17.1%) 166.1048 (12.5%) 176.0882 (100%) 190.1047 (62.0%) 204.1202 (16.1%) 218.1355 (7.8%) 246.1658 (8.7%) 260.1833 (9.5%) 288.2130 (2.8%) 302.2281 (1.6%) 558.4120 (11.4%) 576.4227 (64.6%) 579.4100 (23.6%) 594.4324 (49.4%)	122.0978 (16.9%) 134.0934 (32.8%) 148.1022 (16.2%) 166.1046 (11.1%) 176.0888 (100%) 190.1038 (65.9%) 204.1195 (14.8%) 218.1361 (6.8%) 246.1661 (9.3%) 260.1832 (8.1%) 288.2132 (2.5%) 302.2278 (1.4%) 558.4122 (12.8%) 576.4228 (62.7%) 579.4110 (29.2%) 594.4337 (51.5%)	122.0970 (20.0%) 134.0930 (18.0%) 148.0985 (10.5%) 166.1039 (15.8%) 176.0888 (69.2%) 190.1042 (28.1%) 204.1201 (10.6%) 218.1350 (4.6%) 246.1669 (8.0%) 260.1817 (2.6%) 301.2218 (5.0%) 302.2257 (1.5%) 315.2399 (1.1%) 545.4051 (9.8%) 560.4275 (100%) 563.4172 (16.4%) 578.4369 (19.7%)	122.0965 (20.9%) 134.0926 (18.7%) 148.0982 (9.7%) 166.1036 (15.9%) 176.0881 (70.3%) 190.1034 (30.3%) 204.1196 (9.9%) 218.1356 (4.1%) 246.1665 (9.7%) 260.1815 (2.8%) 301.2225 (4.1%) 302.2278 (1.5%) 315.2379 (1.2%) 545.4049 (10.6%) 560.4284 (100%) 563.4182 (14.8%) 578.4393 (21.0%)	134.0863 (17.7%) 148.1041 (21.4%) 162.1281 (14.1%) 166.1037 (8.9%) 176.0879 (100%) 190.1035 (10.5%) 204.1192 (13.4%) 218.1345 (12.5%) 232.1499 (2.5%) 260.1827 (4.6%) 274.1980 (1.7%) 288.2128 (1.8%) 315.2367 (10.1%) 316.2420 (4.6%) 330.2585 (1.0%) 560.4597 (0.8%) 563.4499 (8.7%) 578.4740 (66.8%)	134.0850 (19.0%) 148.1041 (20.1%) 162.1273 (15.8%) 166.1032 (7.7%) 176.0875 (100%) 190.1032 (8.7%) 204.1196 (13.5%) 218.1350 (15.0%) 232.1492 (3.9%) 260.1815 (5.6%) 274.1990 (1.3%) 288.2135 (2.6%) 315.2368 (8.6%) 316.2437 (3.7%) 330.2603 (1.1%) 560.4602 (0.6%) 563.4492 (7.9%) 578.4756 (63.1%)

### Compound 13 was identified as a completely novel BR 6-deoxo-28-homoTY

The chemical structures of the remaining candidates were also deduced from their MS and MS/MS characteristics (Table 2). However, limited by the availability of commercial authentic standards, the proposed structures could not be confirmed. Note compound 9 and compound 13. Compound 9 was predicted to be 6-deoxo-28-norTY or 6-deoxo-28-norTE, both of which are important intermediates on the C_27_ BRs biosynthetic pathway (Figures 4D, 5D, Supplementary Material).

**Figure 7:**
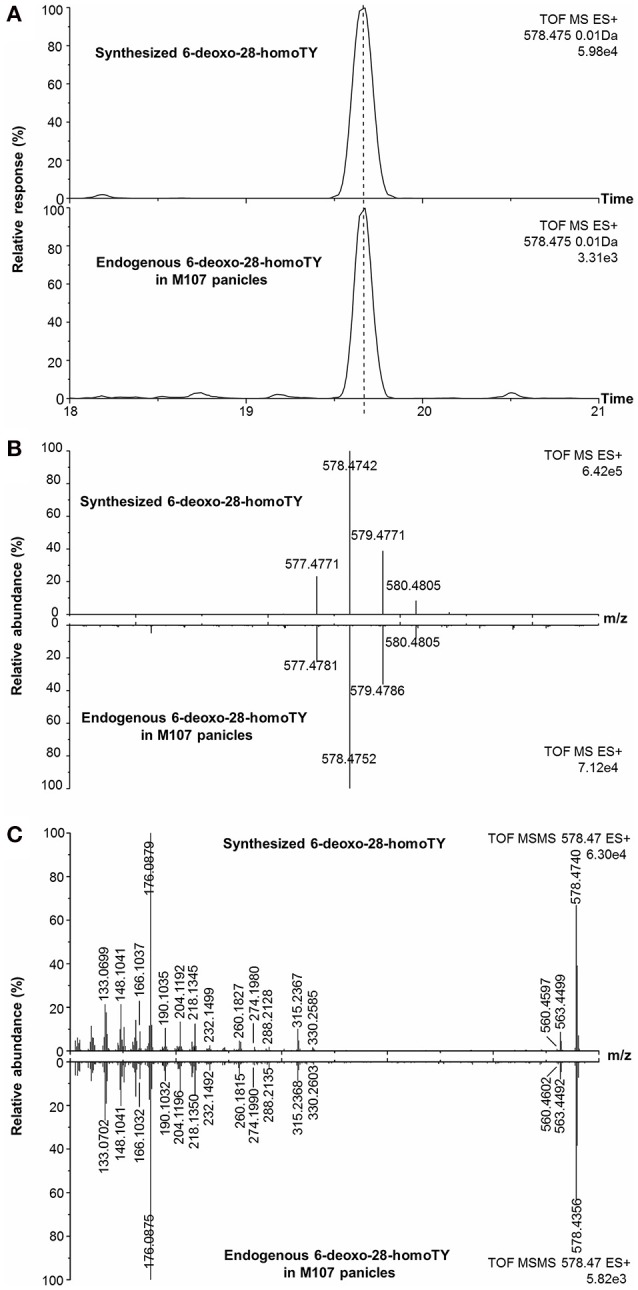
**Identification of endogenous 6-deoxo-28-homoTY in plant tissues with synthetic standard. (A)** EICs of endogenous compound 13 and synthesized 6-deoxo-28-homoTY; **(B)** Isotopic pattern of endogenous compound 13 and synthesized 6-deoxo-28-homoTY; **(C)** High-resolution MS/MS spectra of endogenous compound 13 and synthesized 6-deoxo-28-homoTY.

With regard to compound 13, the measured m/z of [M+H]^+^ was 578.4752 and the molecular formula was calculated to be C_37_H_60_BNO_3_ (theoretical m/z = 578.4745, 0.5 ppm) (Figure 7B). After deducting the contribution of the derivatization reagent DMAPBA, the native formula was calculated to be C_29_H_52_O_3_, which was different with any BR compounds previously reported. The [M+H]^+^ peaks showed an obvious characteristic boron isotopic abundance distributions, which were 21.52, 100, and 36.15% compared to the theoretical distribution (22.54, 100, and 39.38% for ^10^B^12^C-C_29_H_52_O_3_, ^11^B^12^C-C_29_H_52_O_3_, and ^11^B^13^C-C_29_H_52_O_3_, respectively) with relative deviations at −4.5, 0, and −8.2% (Figure 7B). This result indicated the existence of the C_22_, C_23_-diol substructure in compound 13. The EPI spectrum contained product ions at m/z 560.4 generated from H_2_O neutral loss, which suggested that only one hydroxyl group was located on the A ring. This result was confirmed by the accurate m/z of the ion obtained via the QTof-MS system, which was measured to be 560.4602 (theoretical m/z = 560.4639, Δm/m = −6.6 ppm) (Figure 7C). The product ions at m/z 315.2, 316.2, 329.2, and 330.2 were much more abundant than 438.3, 452.3 in the MS/MS spectra (Figures 4G, 7C). This result, combined with the molecular formula information, the hydroxyl group number on the A ring, and the side chain, suggested that the B ring of this compound was 6-deoxo-structured. It could also be seen from the EPI spectrum and the high resolution MS/MS spectrum that both of them contained the ion series from m/z 190.10 to 302.22, a typical MS/MS characteristic of the BRs side chain. In addition, the S/N ratios of [M+H]^+^ > 438.3, 452.4, were at least 4.3-fold higher than [M+H]^+^ > 410.4, 424.4, providing further evidence for the C_29_ structure of the compound (Table 2). To summarize from these MS-based points, compound 13 was a C_29_ 6-deoxo-type BR and could be easily proposed to be either 6-deoxo-28-homoTY or 6-deoxo-28-homoTE, the 28-homoBL biosynthetic precursors corresponding to 6-deoxo-TY or 6-deoxo-TE in C_28_ BRs biosynthesis, respectively. Most importantly, both 6-deoxo-28-homoTY and 6-deoxo-28-homoTE are novel and previously undescribed plant endogenous metabolites and have not been reported elsewhere. Therefore, the discovery of compound 13 is significant for the successful completion of the C_29_ BRs biosynthesis network.

**Figure 8:**
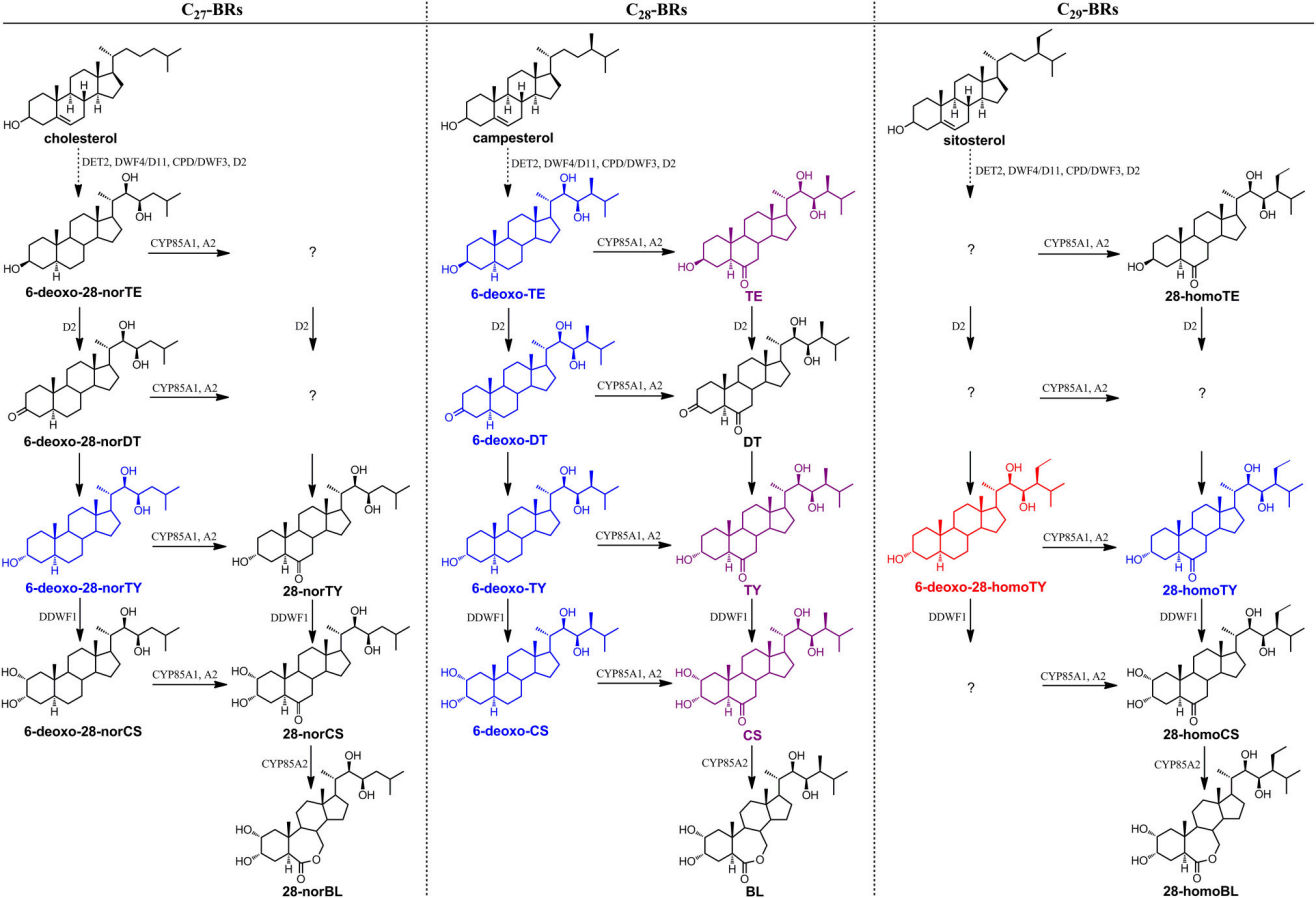
**Stereochemical structure of 6-deoxo-28-homoTY and its location in the BRs biosynthetic network**. This depiction shows only the important C_22, 23_-diol BRs intermediates in the network. The red-colored structure is 6-deoxo-28-homoTY, first discovered and identified in plants. The purple-colored compounds are also found and identified in rice using the described screening method; however, these have been reported before. The blue-colored compounds are found using this method but not identified.

Furthermore, in order to clarify the stereochemical structure of compound 13, we chemically synthesized both 6-deoxo-28-homoTY and 6-deoxo-28-homoTE (Supplementary Material). Through a detailed comparison, the retention time, MS, and MS/MS behavior of compound 13 revealed a full match with 6-deoxo-28-homoTY, rather than with 6-deoxo-28-homoTE (Figures 7A–C, Table 3). Ultimately, compound 13 was identified as 6-deoxo-28-homoTY (Figure 8). As shown in the BRs synthetic network (Figure 8), the discovery of 6-deoxo-28-homoTY filled a blank and is the first reported intermediate in the late C-6 oxidation pathway of C_29_ BRs.

To summarize, using the MS-based screening and identification method, 14 BR candidates were explored, covering C_27_, C_28_, and C_29_ BRs. For C_27_ and C_29_ BRs in particular, there still remain numerous compound vacancies in the early C6-oxidation pathway of C_27_ BRs and in the late C-6 oxidation pathway of C_29_ BRs (Figure 8). The strategy presented here provides a powerful alternative for the traditional BRs discovery method and will facilitate identification of key intermediates in plants, thus improving BRs biosynthesis research.

### Confirmation of 14 BR candidates in BR deficient mutants

**Figure 9:**
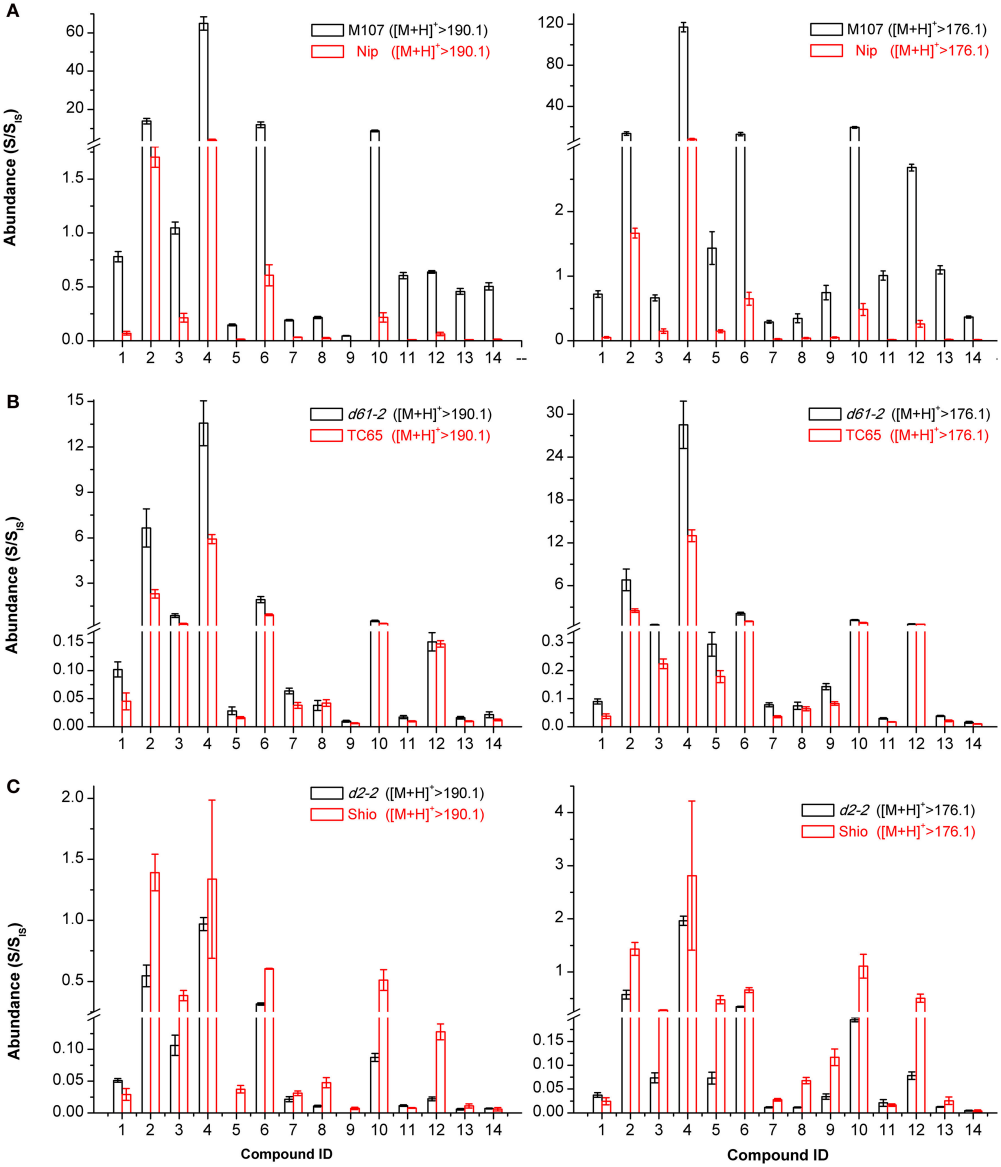
**The quantitative levels of 14 BR candidates in rice BR mutants and their wild types**. Nip **(A)**, Shio **(B)**, and *TC65* **(C)** are wild type plants for M107 **(A)**, *d2-2* **(B)**, and *d61-2* **(C)** mutants, respectively. Samples were analyzed with three technical replicates from sample preparation to LC-MS/MS analysis. The compound abundances in plants were evaluated by the ratio of the integrated peak area of these compounds to that of the spiked D-labeled internal standards (S/S_IS_). The endogenous levels were quantified using two ion channels [M+H]^+^ > 190.1 and [M+H]^+^ > 176.1. Error bars represent the SD.

If the candidates that were screened out were in fact BRs, the endogenous levels of these compounds involved in the BRs biosynthesis and metabolism network would be very likely to be regulated by key genes of the BRs biosynthetic and signaling pathways. Therefore, we measured and compared the contents of these BR candidates in panicles of rice BR-related mutants M107, *d61-2*, and *d2-2* as well as their corresponding wild types. The closer one in retention time of D_3_-6-deoxo-24-epiCS and D_3_-CS indicating similar physicochemical properties was then used as the IS to obtain quantitative information of a certain compound. The M107 mutant is a dominant mutant with enhanced expression of CYP724B1/D11 enzyme catalyzing the C_22_ hydroxylation of steroids, a rate-limiting step in BR biosynthetic pathway (Tanabe et al., 2005; Ohnishi et al., 2006; Wan et al., 2009). Consequently, if the candidates lay on the pathway, accumulated levels would be observed in M107 compared to its wild type Nip. Figure 9A reveals that the endogenous level of all 14 candidates increased by more than 2-fold in M107 with some individuals reaching 10-fold (*p* < 0.01, *t*-test), which well matched the genetic background. The *d61-2* mutant has been reported to be less sensitive to BRs and produce more endogenous BRs due to defects in BR receptor (Yamamuro et al., 2000). Figure 9B clearly shows significant upregulation of the endogenous levels of all 14 candidates (*p* < 0.01) compared to its wild type *TC-65* except for the endogenous levels of compound 8 and 12, that were not significantly increased (*p* > 0.05) according to Student's *t*-test. The *D2* gene encoded the enzyme D2/CYP90D2, which catalyzed the biosynthesis steps of BR C_3_ oxidation, including the reactions converting 6-deoxoTE to 6-deoxo-3-dehydroteasterone (6-deoxo-DT) in the late C-6 oxidation pathway and TE to DT in the early C-6 oxidation pathway. The *d2-2* mutant lost the function of CYP90D2 and the biosynthesis of BR-type compounds on the downstream of 6-deoxoTE and TE was blocked (Hong et al., 2003). Figure 9C shows that the compounds 2, 3, 4, 6, 7, 9, 10, 12, and 13, whose structures were clearly demonstrated, were significantly less biosynthesized (*p* < 0.01, *t*-test) in *d2-2* compared to its wild type Shio which was consistent with their locations on the pathway. For compounds 1, 5, 8, and 14, endogenous levels in the *d2-2* mutant are not as meaningful as others, because their structures are not completely clear and subsequently their locations on the pathway remain unclear. The content of compound 11 was up-regulated in *d2-2*; however, its structure was interpreted as 6-deoxo-DT, which was proposed to be down-regulated. This result was confusing to some extent, possibly due to the use of D_3_-6-deoxo-24-epiCS as its IS for quantification or some unknown regulation mechanisms on 6-deoxo-DT level. In summary, the comparative quantitative information in mutants that are defective in BR biosynthesis and signaling transduction indicated these candidates to be endogenous intermediates on BR biosynthetic pathway and the screening and identification method was effective and successful. More importantly, the endogenous levels of compound 13 in M107, *d61-2*, and *d2-2* mutants suggest 6-deoxo-28-homoTY to be a likely C_29_ BR precursor, which strongly supports our screening and identification results.

In conclusion, we have presented a novel screening and identification strategy for bioactive BRs based on comprehensive UPLC-MS techniques. The correlation between the MS, MS/MS characteristics of BRs and their structures has been established first. Furthermore, 14 potential BRs were discovered and structurally elucidated by following a MS-based clue acquired through MRM-dependent EPI, high resolution MS, and MS survey-dependent MS/MS. It is notable that a totally new C_29_ BR identified as 6-deoxo-28-homoTY was discovered and reported for the first time. With the discovery of more BR biosynthetic and signaling mutants, this method promises a viable way to find additional chemically new BR biosynthetic intermediates, which will be significant to further our understanding of the biosynthesis and metabolism network of BRs. The strategy presented here is also promising for research on other plant hormones, such as CKs and SLs.

## Author contributions

PX, JC, and CY designed the research. PX, JY, and BL performed the experiments. PX processed the data. PX and JC wrote the paper. SF and JF participated in data processing. HT, YS, and WT chemically synthesized 6-deoxo-28-homo TY and 6-deoxo-28-homoTE.

## Funding

This work was supported by the National Natural Science Foundation of China (grant nos. 31500299, 31470433, and 91417314) and the CAS Key Technology Talent Program.

### Conflict of interest statement

The authors declare that the research was conducted in the absence of any commercial or financial relationships that could be construed as a potential conflict of interest.

## References

[B1] Al-Babili S. Bouwmeester H. J. (2015). Strigolactones, a novel carotenoid-derived plant hormone. Annu. Rev. Plant Biol. 66, 161–186. 10.1146/annurev-arplant-043014-114759 25621512

[B2] Bajguz A. (2009). Isolation and characterization of brassinosteroids from algal cultures of *Chlorella vulgaris* Beijerinck (Trebouxiophyceae). J. Plant Physiol. 166, 1946–1949. 10.1016/j.jplph.2009.05.003 19535168

[B3] Fujioka S. Inoue T. Takatsuto S. Yanagisawa T. Yokota T. Sakurai A. (1995). Identification of a new brassinosteroid, cathasterone, in cultured cells of *Catharanthus roseus* as a biosynthetic precursor of teasterone. Biosci. Biotechnol. Biochem. 59, 1543–1547. 10.1271/bbb.59.1543

[B4] Fujioka S. Noguchi T. Takatsuto S. Sakurai A. Yoshida S. Li J. M.. (1999). Arabidopsis det2 is defective in the conversion of (24R)-24-methylcholest-4-en-3-one to (24R)-24-methyl-5α-cholestan-3-one in brassinosteroid biosynthesis. Plant Physiol. 120, 833–839. 10.1104/pp.120.3.833 10398719 PMC59322

[B5] Fujioka S. Yokota T. (2003). Biosynthesis and metabolism of brassinosteroids. Annu. Rev. Plant Biol. 54, 137–164. 10.1146/annurev.arplant.54.031902.134921 14502988

[B6] Fujita S. Ohnishi T. Watanabe B. Yokota T. Takatsuto S. Fujioka S.. (2006). Arabidopsis CYP90B1 catalyses the early C-22 hydroxylation of C_27_, C_28_ and C_29_ sterols. Plant J. 45, 765–774. 10.1111/j.1365-313X.2005.02639.x 16460510

[B7] Grove M. D. Spencer G. F. Rohwedder W. K. Mandava N. Worley J. F. Warthen J. D. Jr. (1979). Brassinolide, a plant growth-promoting steroid isolated from *Brassica napus* pollen. Nature 281, 216–217.

[B8] Hayat S. (2011). Brassinosteroids: A Class of Plant Hormone. Dordrecht: Springer Science+Business Media.

[B9] Hoff R. B. Meneghini L. Pizzolato T. N. M. Peralba M. D. C. R. Díaz-Cruz M. S. Barceló D. (2014). Structural elucidation of sulfaquinoxaline metabolism products and their occurrence in biological samples using high-resolution orbitrap mass spectrometry. Anal. Chem. 86, 5579–5586. 10.1021/ac501132r 24796379

[B10] Hong Z. Ueguchi-Tanaka M. Umemura K. Uozu S. Fujioka S. Takatsuto S.. (2003). A rice brassinosteroid-deficient mutant, ebisu dwarf (d2), is caused by a loss of function of a new member of cytochrome P450. Plant Cell 15, 2900–2910. 10.1105/tpc.014712 14615594 PMC282825

[B11] Hostettmann K. Wolfender J. L. Terreaux C. (2001). Modern screening techniques for plant extracts. Pharm. Biol. 39, 18–32. 10.1076/phbi.39.s1.18.0008 21554168

[B12] Hwang J. Y. Park C. H. Namgung H. Kim S. K. (2006). Identification of a new brassinosteroid, 23-dehydro-2-epicastasterone, from immature seeds of *Phaseolus vulgaris*. J. Plant Biol. 49, 409–412. 10.1007/BF03178820

[B13] Joo S.-H. Jang M.-S. Kim M. K. Lee J.-E. Kim S.-K. (2015). Biosynthetic relationship between C28-brassinosteroids and C29-brassinosteroids in rice (*Oryza sativa*) seedlings. Phytochemistry 111, 84–90. 10.1016/j.phytochem.2014.11.006 25433632

[B14] Kim B. K. Fujioka S. Takatsuto S. Tsujimoto M. Choe S. (2008). Castasterone is a likely end product of brassinosteroid biosynthetic pathway in rice. Biochem. Biophys. Res. Commun. 374, 614–619. 10.1016/j.bbrc.2008.07.073 18656444

[B15] Kim S.-K. Abe H. Little C. A. Pharis R. P. (1990). Identification of two brassinosteroids from the cambial region of Scots pine (*Pinus silverstris*) by gas chromatography-mass spectrometry, after detection using a dwarf rice lamina inclination bioassay. Plant physiol. 94, 1709–1713. 10.1104/pp.94.4.1709 16667906 PMC1077442

[B16] Mitchell J. W. Mandava N. Worley J. F. Plimmer J. R. Smith M. V. (1970). Brassins—a new family of plant hormones from rape pollen. Nature 225, 1065–1066. 10.1038/2251065a0 16056912

[B17] Ni S. Qian D. Duan J.-A. Guo J. Shang E.-X. Shu Y.. (2010). UPLC–QTOF/MS-based screening and identification of the constituents and their metabolites in rat plasma and urine after oral administration of *Glechoma longituba* extract. J. Chromatogr. B Analyt. Technol. Biomed. Life Sci. 878, 2741–2750. 10.1016/j.jchromb.2010.08.014 20829126

[B18] Ohnishi T. Godza B. Watanabe B. Fujioka S. Hategan L. Ide K.. (2012). CYP90A1/CPD, a brassinosteroid biosynthetic cytochrome P450 of *Arabidopsis*, catalyzes C-3 oxidation. J. Biol. Chem. 287, 31551–31560. 10.1074/jbc.M112.392720 22822057 PMC3438987

[B19] Ohnishi T. Watanabe B. Sakata K. Mizutani M. (2006). CYP724B2 and CYP90B3 function in the early c-22 hydroxylation steps of brassinosteroid biosynthetic pathway in tomato. Biosci. Biotechnol. Biochem. 70, 2071–2080. 10.1271/bbb.60034 16960392

[B20] Quadri S. S. Stratford R. E. Boué S. M. Cole R. B. (2013). Screening and identification of glyceollins and their metabolites by electrospray ionization tandem mass spectrometry with precursor ion scanning. Anal. Chem. 85, 1727–1733. 10.1021/ac3030398 23294002 PMC3593975

[B21] Sakakibara H. (2006). Cytokinins: activity, biosynthesis, and translocation. Annu. Rev. Plant Biol. 57, 431–449. 10.1146/annurev.arplant.57.032905.105231 16669769

[B22] Sakamoto T. Morinaka Y. Ohnishi T. Sunohara H. Fujioka S. Ueguchi-Tanaka M.. (2006). Erect leaves caused by brassinosteroid deficiency increase biomass production and grain yield in rice. Nat. Biotechnol. 24, 105–109. 10.1038/nbt1173 16369540

[B23] Tanabe S. Ashikari M. Fujioka S. Takatsuto S. Yoshida S. Yano M.. (2005). A novel cytochrome P450 is implicated in brassinosteroid biosynthesis via the characterization of a rice dwarf mutant, dwarf11, with reduced seed length. Plant Cell 17, 776–790. 10.1105/tpc.104.024950 15705958 PMC1069698

[B24] Vriet C. Russinova E. Reuzeau C. (2012). Boosting crop yields with plant steroids. Plant Cell 24, 842–857. 10.1105/tpc.111.094912 22438020 PMC3336137

[B25] Wan S. Wu J. Zhang Z. Sun X. Lv Y. Gao C.. (2009). Activation tagging, an efficient tool for functional analysis of the rice genome. Plant Mol. Biol. 69, 69–80. 10.1007/s11103-008-9406-5 18830797

[B26] Wen B. Ma L. Nelson S. D. Zhu M. (2008). High-throughput screening and characterization of reactive metabolites using polarity switching of hybrid triple quadrupole linear ion trap mass spectrometry. Anal. Chem. 80, 1788–1799. 10.1021/ac702232r 18251522

[B27] Xin P. Yan J. Fan J. Chu J. Yan C. (2013a). A dual role of boronate affinity in high-sensitivity detection of vicinal diol brassinosteroids from sub-gram plant tissues via UPLC-MS/MS. Analyst 138, 1342–1345. 10.1039/c3an36533f 23340859

[B28] Xin P. Y. Yan J. J. Fan J. S. Chu J. F. Yan C. Y. (2013b). An improved simplified high-sensitivity quantification method for determining Brassinosteroids in different tissues of rice and Arabidopsis. Plant Physiol. 162, 2056–2066. 10.1104/pp.113.221952 23800992 PMC3729782

[B29] Yamamuro C. Ihara Y. Wu X. Noguchi T. Fujioka S. Takatsuto S.. (2000). Loss of function of a rice brassinosteroid insensitive1 homolog prevents internode elongation and bending of the lamina joint. Plant Cell 12, 1591–1605. 10.1105/tpc.12.9.1591 11006334 PMC149072

[B30] Yan Z. Lin G. Ye Y. Wang Y. Yan R. (2014). Triterpenoid saponins profiling by adducts-targeted neutral loss triggered enhanced resolution and product ion scanning using triple quadrupole linear ion trap mass spectrometry. Anal. Chim. Acta 819, 56–64. 10.1016/j.aca.2014.02.019 24636411

