# Supplementary material for: A Comprehensive and Effective Mass Spectrometry-Based Screening Strategy for Discovery and Identification of New Brassinosteroids from Rice Tissues
Source: Front Plant Sci. 2016 Nov 30;7:1786. doi: 10.3389/fpls.2016.01786 (PMC5127834; doi:10.3389/fpls.2016.01786)
Supplement: Supplementary file 1 [file DataSheet1.DOCX]

**Supplementary Material for**

**A Comprehensive and Effective Mass Spectrometry-Based Screening Strategy for Discovery and Identification of New Brassinosteroids from Rice Tissues**

**Figure S1. EPI spectrum of** **candidate compound 4**


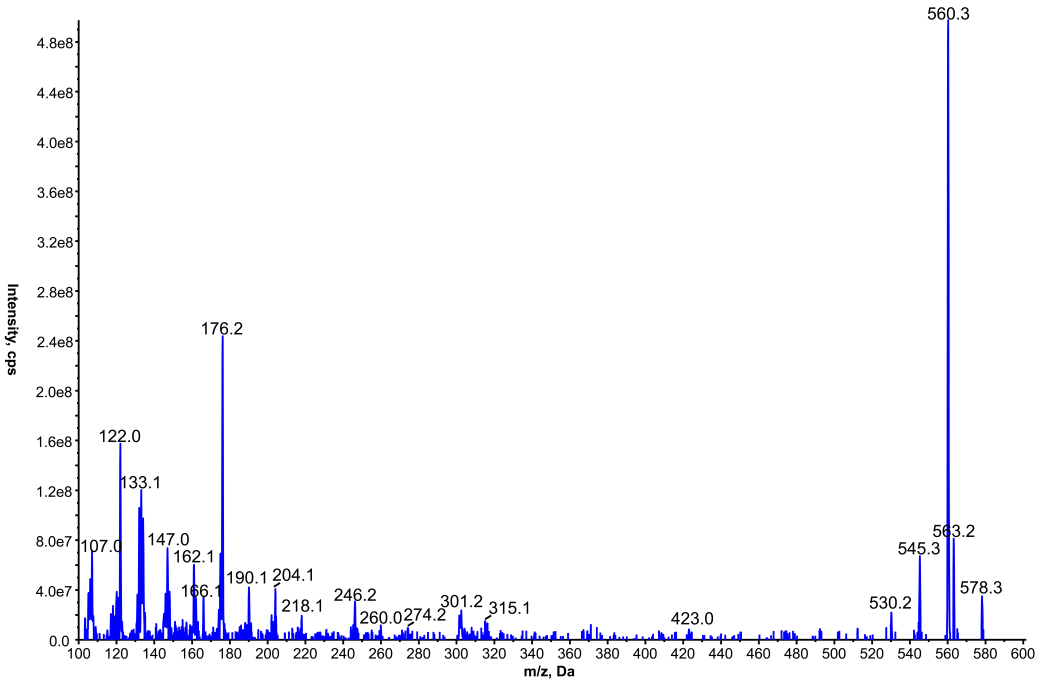


**Figure S2. EPI spectrum of candidate compound 6**


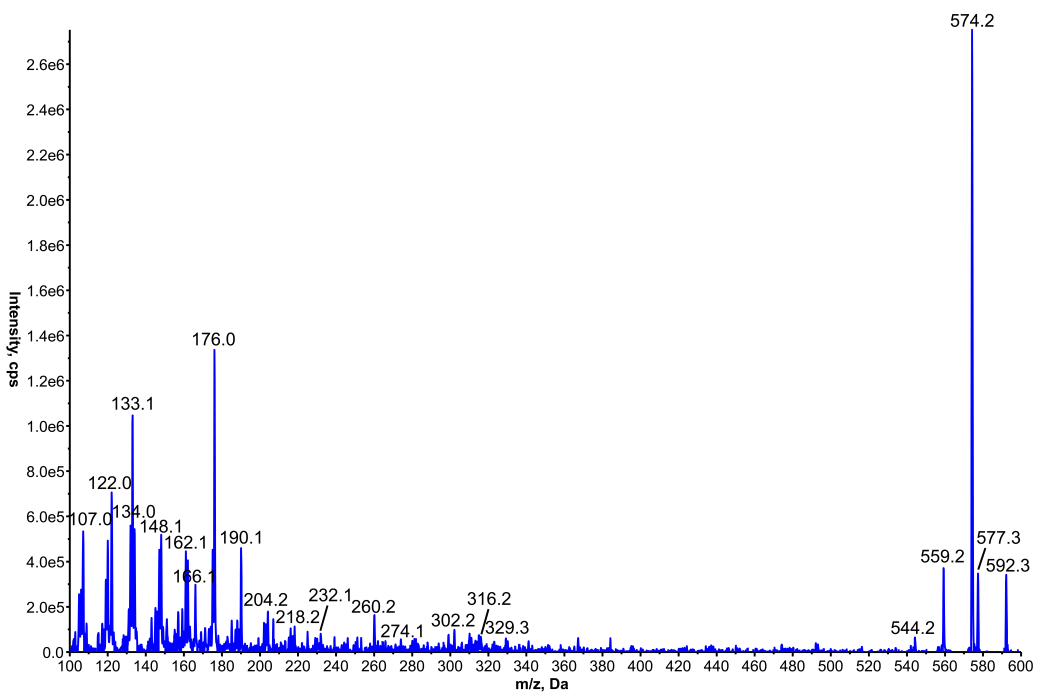


**Figure S3. HR MS/MS spectrum of candidate compound 6**


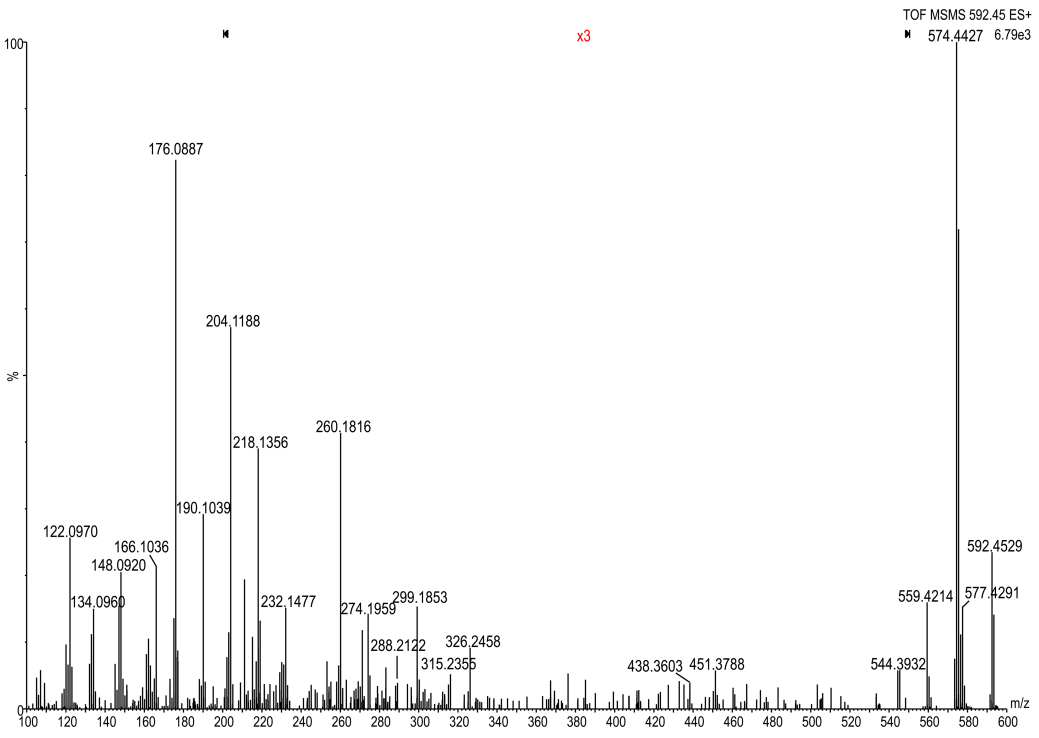


**Figure S4. Confirmation of candidate compound 4 in plant tissues as TY with authentic reference standard.** (A) EICs of endogenous TY and TY reference standard; (B) Isotopic pattern of endogenous TY and TY reference standard; (C) High resolution MS/MS spectra of endogenous TY and TY reference standard.


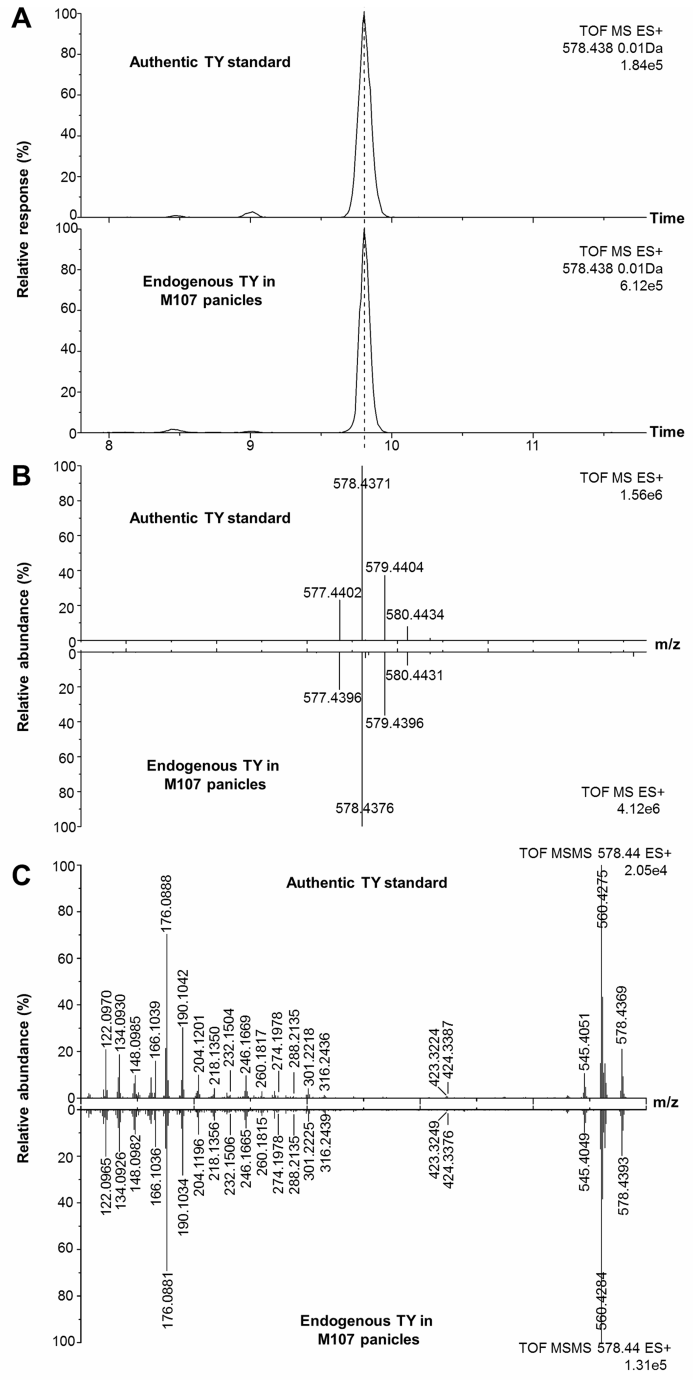


**Structural elucidation of compound 9**

The measured m/z of [M+H]^+^ was 550.4432 and the molecular formula was calculated to be C_35_H_56_BNO_3_ (theoretical m/z=550.4432, 0 ppm). After deducting the contribution of the derivatization reagent DMAPBA, the native formula was calculated to be C_27_H_48_O_3_, which is same to 6-deoxo-28-norTY. The [M+H]^+^ peaks showed an obvious characteristic boron isotopic abundance distribution, which is 22.07%, 100%, 37.28% compared to the theoretical distribution (22.65%, 100% and 37.365% for ^10^B^12^C-BRs, ^11^B^12^C-BRs and ^10^B^13^C-BRs, respectively) with relative deviations at -2.56%, 0, -0.23%. This result indicated the existence of C_22_, C_23_-diol substructure in compound 9. The EPI spectrum contained product ions at m/z 532.4 generated from H_2_O neutral losses, which suggested that only one hydroxyl groups were located on A ring. This result was confirmed by the accurate m/z measured with QTof-MS system, which was measured to be 532.4351 (theoretical m/z=576.4326, ∆m/m=4.69 ppm). The product ions at m/z 301.2, 302.2, 287.1, 288.1 was much more abundant than 410.3, 424.3 in the MS/MS spectra. This phenomenon, combined with the molecular formula information and the hydroxyl group number on A ring and the side chain, could easily lead to such a judgment that B ring of this compound was a 6-deoxo- C_27_ structured. It could also be seen from the EPI spectrum and the high resolution MS/MS spectrum both of them contained the ion series from m/z 190.10 to 274.20, a typical MS/MS characteristic of BRs side chain. In addition, the S/N ratios of [M+H]^+^>410.4, 424.4 were 7 and 6, while [M+H]^+^>438.3, 452.4 channels were not detectable, which provided another evidence for that the compound was C_27_ structured. To summarize from these MS-based information, compound 9 was a C_27_ 6-deoxo-type BR with one hydroxyl on A ring and could be easily recognized to be 6-deoxo-28-norTY or 6-deoxo-28-norTE. However, neither 6-deoxo-28-norTY nor 6-deoxo-28-norTE was commercially available, the structure of compound 9 couldn’t be totally determined.

**Experimental Procedures and Characterization Data for The synthesis of 6-deoxo-28-homo-typhasterol (6-deoxo-28-homoTY) from stigmasterol**

6-deoxo-28-homoTY (**8**) can be synthesized from commercially available stigmasterol (**1**) (Scheme S1). Oppenauer oxidation of **1** resulted in the formation of stigmast-4, 22-dien-3-one **2**, the conjugated reduction of which was achieved via Birch reduction, giving the AB *trans*-fused 5α-stigmast-22-en-one **3**.[^1^](#_ENREF_1) Reduction of **3** with NaBH_4_ provided 3S-4(5)-dihydro-5α-stigmasterol (**4**) stereoselectively. Through Sharpless asymmetric dihydroxylation (AD-mix β) 5α-stigmasterol (**4**) is converted to corresponding 3R-6-deoxo-28-homoTY **5,** an C_3_-epimer of 6-deoxo-28-homoTY also called 6-deoxo-28-homoTE.[^2^](#_ENREF_2) Inversion of the 3β-OH group in **4** by 4-nitrobenzoic acid under standard Mitsunobu conditions gave 3R-4(5)-dihydro-5α-stigmasterol **(6)** in good yield. Sharpless asymmetric dihydroxylation (ADmix-β) of **6** followed by a methanolysis of the resulting **7**, gave the desired 6-deoxo-24-homoTY (**8)**. The structures of 6-deoxo-28-homoTY (**8**) and its C3-epimer **5** are assigned according to reaction conditions and the comparison of their spectrum with that of known brassinolide and analogues.

**Scheme S1. Synthesis of 6-deoxo-28-homoTY (8) and its C_3_-epimer 6-deoxo-28-homoTE (5) from stigmasterol (1)**

**General Methods:** All reactions sensitive to air or moisture were performed in flame-dried round bottom flasks with rubber septum under a positive pressure of argon or nitrogen atmosphere, unless otherwise noted. Air and moisture-sensitive liquids and solutions were transferred via syringe and stainless steel cannula. Tetrahydrofuran (THF) were distilled from sodium/benzophenone, methylene chloride (DCM), toluene from calcium hydride and others according to the standard procedures described in *Purification of* *Laboratory Chemicals* (2009). Yields refer to chromatographically and spectroscopically (^1^HNMR) homogeneous materials, unless otherwise stated. Reactions were monitored by thin layer chromatography (TLC) carried out on silica gel plates using UV light as visualizing agent and an ethanolic solution of phosphomolybdic acid, and heat as developing agents. NMR spectra were recorded on Bruker DRX-400 instrument and calibrated using residual undeuterated solvent as an internal reference [^1^H NMR: CHCl_3_ (7.26), C_6_HD_5_ (7.16), DMSO-*d_6_* (2.50); ^13^C NMR: CDCl_3_ (77.16), C_6_D_6_ (128.06)]. The following abbreviations were used to explain the multiplicities: s = singlet, d = doublet, t = triplet, q = quartet, br = broad.

**Stigmasta-4,22-dien-3-one (2)**: A solution of stigmasterol **1** (9.0 g, 21.8 mmol) and cyclohexanone (50 mL) in toluene (100 mL) was heated at reflux with a standard Dean–Stark setup for 1 h. To the mixture was added Al(*i*OPr)_3_ (8.0 g, 39 mmol, 1.8 equiv), and the resulting mixture was stirred at reflux for another 4 h. The mixture was cooled, added a diluted HCl solution (1.2 N, 100 mL), and separated. The organic layer was washed with brine, dried over Na_2_SO_4_, filtered, and purified through flash column chromatography on silica gel to give **2** (5.0 g, 56%) as a white solid.[^1^](#_ENREF_1) mp 110–111 °C; ^1^H NMR (400 MHz, CDCl_3_) δ 5.72 (s, 1H), 5.14 (dd, *J* = 15.0, 8.6 Hz, 1H), 5.02 (dd, *J* = 15.2, 8.6 Hz, 1H), 2.22–2.47 (m, 4H), 1.98–2.08 (m, 3H), 1.18 (s, 3H), 1.02 (d, *J* = 6.0 Hz, 3H), 0.78–0.86 (9H), 0.72 (s, 3H); ^13^C NMR (100 MHz, CDCl_3_) δ 199.8, 171.8, 138.3, 129.6, 123.9, 56.1, 56.0, 54.0, 51.4, 42.4, 40.6, 39.7, 38.8, 35.8, 35.8, 34.1, 33.1, 32.2, 32.0, 29.0, 25.5, 24.4, 21.3, 21.2, 21.2, 19.1, 17.5, 12.4, 12.3; ESI-MS *m/z*: 411.3 ([M+H]^+^).

**4(5)-dihydro-5α-Stigmasta-3-one (3)**: To a mixture of THF (50 mL) and ammonia (100 mL) was added Lithium (0.50 g) at -78 °C under argon. The resulting mixture was stirred for 1 h and a solution of **2** (3.5 g, 8.5 mmol) in *t*-BuOH/THF (10 mL/50 mL) was added slowly. The solution was stirred for 2 h and quenched with NH_4_Cl. The ammonia was allowed to evaporate at ambient temperature. The residue was diluted with a saturated aqueous NH_4_Cl solution and extracted with ethyl acetate for three times. The combined organic layers were washed with brine, dried over Na_2_SO_4_, filtered, and purified through flash column chromatography on silica gel to give **3** (2.1 g, 56%) a pale yellow solid.[^1^](#_ENREF_1) mp: 148–149 °C; ^1^H NMR (400 MHz, CDCl_3_) δ 5.15 (dd, *J* = 15.0, 8.2 Hz, 1H), 5.01 (dd, *J* = 15.0, 8.6 Hz, 1H), 2.22–2.43 (m, 3H), 1.94–2.10 (m, 4H), 1.01 (s, 3H), 1.01 (d, *J* = 6.6 Hz, 3H), 0.77–0.86 (9H), 0.69 (s, 3H); ^13^C NMR (100 MHz, CDCl_3_) δ 212.4, 138.4, 129.4, 56.5, 56.2, 53.9, 51.4, 46.8, 44.9, 42.6, 40.7, 39.9, 38.7, 38.4, 35.8, 35.5, 32.0, 31.9, 29.1, 25.6, 24.4, 21.6, 21.3, 21.3, 19.1, 12.4, 12.4, 11.6; ESI-MS *m/z*: 413.3 ([M+H]^+^).

**4(5)-dihydro-5α-Stigmasta-3β-ol** (**4**): A solution of ketone **3** (1.5 g, 3.6 mmol) in MeOH/THF (20 mL/10 mL) was treated with NaBH_4_ (152 mg, 4.0 mmol, 1.1 equiv) at ambient temperature for 2 h, and was quenched with HOAc (1 mL). The mixture was concentrated under reduced pressure and purified through flash column chromatography on silica gel to provide **4** (1.20 g, 80%) as a white solid.[^3^](#_ENREF_3)^,^ [^4^](#_ENREF_4) mp: 128 °C; ^1^H NMR (400 MHz, CDCl_3_) δ 5.08 (dd, *J* = 15.0, 8.6 Hz, 1H), 4.93 (dd, *J* = 15.0, 8.3 Hz, 1H), 3.94–3.99 (m, 1H), 1.84–2.01 (m, 2H), 0.94 (d, *J* = 7.2 Hz, 3H), 0.70–0.79 (12H), 0.60 (s, 3H); ^13^C NMR (100 MHz, CDCl_3_) δ 138.5, 129.3, 71.5, 56.8, 56.2, 54.5, 51.4, 45.0, 42.6, 40.7, 40.1, 38.4, 37.2, 35.7, 35.6, 32.2, 32.0, 31.7, 29.1, 28.9, 25.6, 24.4, 21.4, 21.3, 21.2, 19.1, 12.5, 12.4, 1.2; ESI-MS *m/z*: 437.3 ([M+Na]^+^).

**3R-6-deoxo-28-homoTY (5)**: To a mixture of **4** (300 mg, 0.72 mmol), (DHQD)_2_PHAL (237 mg, 0.3 mmol, 0.4 equiv), K_3_Fe(CN)_6_ (1.40 g, 4.2 mmol, 6 equiv), MsNH_2_ (138 mg, 1.45 mmol, 2 equiv), and K_2_CO_3_ (0.60 g, 4.3 mmol, 6 equiv) in H_2_O/*t*-BuOH/THF (1/1/1, 12 mL) was added K_2_OsO_4_•2H_2_O (127 mg, 0.073 mmol, 0.1 equiv). The reaction mixture was stirred at ambient temperature for 7 days and then quenched with sodium sulfite. Stirring was continued for 1 h and the solution extracted with CH_2_Cl_2_/MeOH (95/5, 3 × 30 mL). The combined organic layers were washed with HCl (1.2 N), NaHCO_3_ (saturated aqueous solution), brine, dried over Na_2_SO_4_, filtered, and concentrated under reduced pressure. The crude product was purified by flash column chromatography on silica gel to provide **5** (80 mg, 25%) as a white solid.[^2^](#_ENREF_2) mp: 185–186 °C; [α]_D_^22^ + 27.8 (*c* 0.10, MeOH); ^1^H NMR (400 MHz, CDCl_3_) δ 3.71 (d, *J* = 8.6 Hz, 1H), 3.60 (d, *J* = 8.6 Hz, 1H), 3.54–3.64 (m, 1H), 0.92–0.99 (m, 9H), 0.89 (d, *J* = 5.2 Hz, 3H), 0.67 (s, 3H); ^13^C NMR (100 MHz, CDCl_3_) δ 74.9, 72.9, 71.5, 56.5, 54.4, 52.8, 46.4, 44.9, 42.6, 40.2, 38.3, 37.1, 35.7, 35.6, 32.1, 31.7, 29.0, 28.8, 28.0, 24.2, 21.4, 19.6, 19.0, 13.6, 12.5, 12.1, 12.1; ESI-MS *m/z*: 471.3 ([M+Na]^+^); HMRS-ESI (*m/z*): [M+NH_4_]^+^ calcd for C_24_H_52_O_3_, 466.4255; found, 466.4253.

**Mitsunobu inversion**:[^5^](#_ENREF_5) Diethyl azodicarboxylate (1.40 mL, 8.9 mmol) was added with stirring to a solution of the alcohol **4** (800 mg, 1.9 mmol), Ph_3_P (2.0 g, 7.6 mmol), and 4-nitrobenzoic acid (1.30 g, 7.8 mmol) at ambient temperature in THF (20 mL). The resulting orange solution was stirred for 12 h at ambient temperature and then quenched with diluted HCl solution and extracted with ethyl acetate. The combined organic layers were washed with brine, dried over Na_2_SO_4_, filtered, and purified through flash column chromatography on silica gel to give **6** (900 mg, 83%) as a white solid.^1^H NMR (400 MHz, CDCl_3_) δ 8.31 (d, *J* = 8.8 Hz, 2H), 8.22 (d, *J* = 8.8 Hz, 2H), 5.29–5.35 (m, 1H), 5.15 (ddd, *J* = 15.0, 8.8, 1.2 Hz), 5.01 (ddd, *J* = 15.0, 8.2, 2.4 Hz, 1H), 1.01 (d, 6.4 Hz), 0.78–0.86 (m, 12H), 0.69 (s, 3H); ESI-MS *m/z*: 481.4 ([M+NH_4_]^+^).

**6-deoxo-28-homoTY (8)**: To a mixture of **6** (450 mg, 0.8 mmol), (DHQD)_2_PHAL (255 mg, 0.4 equiv), K_3_Fe(CN)_6_ (1.60 g, 6 equiv), MsNH_2_ (155 mg, 2 equiv), and K_2_CO_3_ (670 mg, 6 equiv) in H_2_O/*t*-BuOH/THF (1/1/1, 30 mL) was added K_2_OsO_4_•2H_2_O (30 mg, 0.1 equiv). The reaction mixture was stirred at ambient temperature for 7 days and then quenched with sodium sulfite. Stirring was continued for 1 h and the solution extracted with CH_2_Cl_2_/MeOH (95/5, 3 × 50 mL). The combined organic layers were washed with HCl (1.2 N), NaHCO_3_ (saturated aqueous solution), brine, dried over Na_2_SO_4_, filtered, and concentrated under reduced pressure. The crude product was dissolved with MeOH (20 mL) and treated with K_2_CO_3_ (200 mg, 1.5 mmol) at ambient temperature for 2 h. The mixture was diluted with water (20 mL) and extracted with EtOAc for three times. The combined organic layers were washed with brine, dried over Na_2_SO_4_, filtered, and concentrated. Purification by flash column chromatography on silica gel to give **8** (100 mg, 28%) as a white solid. mp: 192–193 °C; [α]_D_^22^ + 20.8 (*c* 0.10, MeOH); ^1^H NMR (400 MHz, CDCl_3_) δ 4.01–4.05 (m, 1H), 3.78 (d, *J* = 8.4 Hz, 1H), 3.66 (d, *J* = 8.4 Hz, 1H), 0.92–0.99 (m, 8H), 0.88 (d, *J* = 6.0 Hz, 3H), 0.77 (s, 3H), 0.66 (s, 3H); ^13^C NMR (100 MHz, cdcl_3_) δ 74.9, 72.9, 66.7, 56.6, 54.4, 52.7, 46.3, 42.6, 40.2, 39.2, 37.1, 36.2, 36.0, 35.7, 32.3, 32.1, 29.1, 28.9, 28.7, 28.0, 24.2, 21.4, 21.0, 19.7, 19.1, 13.6, 12.1, 12.1, 11.3; ESI-MS *m/z*: 471.3 ([M+Na]^+^); HMRS-ESI (*m/z*): [M+NH_4_]^+^ calcd for C_24_H_52_O_3_, 466.4255; found, 466.4255.

**Reference**:

1. Shu, Y., Jones, S.R., Kinney, W.A. & Selinsky, B.S. The synthesis of spermine analogs of the shark aminosterol squalamine. *Steroids* **67**, 291-304 (2002).

2. Acebedo, S.L., Alonso, F., Ramírez, J.A. & Galagovsky, L.R. Synthesis of aromatic stigmastanes: application to the synthesis of aromatic analogs of brassinosteroids. *Tetrahedron* **68**, 3685-3691 (2012).

3. Barrero, A.F., Sanchez, J.F., Alvarez-Manzaneda, E.J., Dorado, M.M. & Haidour, A. Terpenoids and sterols from the wood of Abies pinsapo. *Phytochemistry* **32**, 1261-1265 (1993).

4. Takatsuto, S. & Ikekawa, N. Synthesis of 6-deoxohomodolichosterone, a new plant-growth-promoting steroid. *Journal of the Chemical Society, Perkin Transactions 1*, 2269-2272 (1986).

5. Martin, S.F., Dodge, J.A., Burgess, L.E., Limberakis, C. & Hartmann, M. Novel approach to the ansamycin antibiotics macbecin I and herbimycin A. A formal total synthesis of (+)-macbecin I. *Tetrahedron* **52**, 3229-3246 (1996).
